# DEAD-box ATPase–marked condensates coordinate compartmentalized translation and antibiotic persistence

**DOI:** 10.1126/sciadv.ady1930

**Published:** 2026-01-02

**Authors:** Ziyin Zhang, Daqian Li, Bo Zheng, Jia-feng Liu

**Affiliations:** ^1^ Center for Infectious Disease Research, School of Basic Medical Sciences, Tsinghua University, Beijing, China.; ^2^ Tsinghua-Peking Center for Life Sciences, Tsinghua University, Beijing, China.

## Abstract

Antibiotic-tolerant persisters use dormancy as a bet-hedging strategy to evade lethal antibiotics, undermining therapeutic efficacy. Protein condensates have been implicated in bacterial dormancy, yet how these assemblies orchestrate dormancy entry remains unclear. We evolved persisters that enter dormancy before the stationary phase, most harboring mutations in *serS*, encoding seryl-transfer RNA synthetase (SerRS). These variants recapitulated persistence induced by serine hydroxamate (SHX), a serine analog and SerRS inhibitor. Both the *serS* mutation and SHX treatment trigger SerRS sequestration into conserved DEAD-box adenosine triphosphatase–associated condensates, coinciding with growth arrest and dormancy. In vitro, the SerRS variant preferentially partitions into DeaD granules, consistent with its distinct in vivo localization. Microscopy revealed spatially restricted translation silencing within condensates upon SerRS partitioning. Together, these phase-separated condensates act as hubs that coordinate the transition from proliferation to dormancy, paralleling eukaryotic cell fate control via localized translation. Our findings provide mechanistic insight into bacterial persistence and suggest that targeting condensates could help combat antibiotic tolerance and delay resistance.

## Teaser

Sequestration of SerRS into condensates silences local translation, promoting bacterial dormancy and antibiotic tolerance.

## INTRODUCTION

Antibiotic-tolerant persistence refers to a small subpopulation of bacteria that endure intensive antibiotic exposure (*1*). By surviving high-dose antibiotics, persisters serve as reservoirs for resistance evolution (*2*–*5*). Bacterial persistence is implicated in many recurrent and chronic infections caused by pathogens, including *Mycobacterium tuberculosis*, *Salmonella enterica*, and *Staphylococcus aureus* (*6*–*9*), undermining antimicrobial efficacy and contributing to antibiotic treatment failure (*5*, *10*–*13*). Given that several bactericidal antibiotics predominantly kill actively growing bacteria, a transient dormant state becomes protective for persisters, shielding them from lethal antibiotic exposure (*1*, *6*, *13*–*16*). Evolutionarily tuned switches between proliferation and dormancy implement a bet-hedging strategy that maximizes bacterial fitness in fluctuating environments (*2*, *17*, *18*).

Recent studies have suggested a correlation between protein condensates and bacterial dormancy (*19*–*23*). These reversible condensates, proposed as markers of bacterial persisters, form under stress conditions and dissolve upon growth resumption, resembling eukaryotic stress granules (*20*, *22*–*24*). However, the mechanisms by which these condensates orchestrate the transition from proliferation to dormancy, thereby facilitating persistence formation, remain to be fully elucidated. Previous investigations have characterized variants evolved from overnight stationary-phase cultures, in which stress-induced mutants exhibit prolonged dormancy when switched to fresh medium (*2*, *25*, *26*). Mutations in aminoacyl-tRNA synthetases (aaRSs) and associated genes (e.g., *metG*, *argS*, *trpS*, *leuS*, *pheS*, *hipA*, and *gltX*) have been linked to this “lag-dependent persistence” (*2*, *25*–*31*). To dissect the transition from cell division to dormancy, we subjected exponentially growing bacterial populations to intermittent antibiotic exposure (*25*), isolating mutants capable of initiating dormancy before the stationary phase and elucidating the role of biomolecular condensates in modulating bacterial persistence in response to environmental fluctuations.

## RESULTS

### Early onset of growth arrest mediates antibiotic persistence

**Fig. 1.**
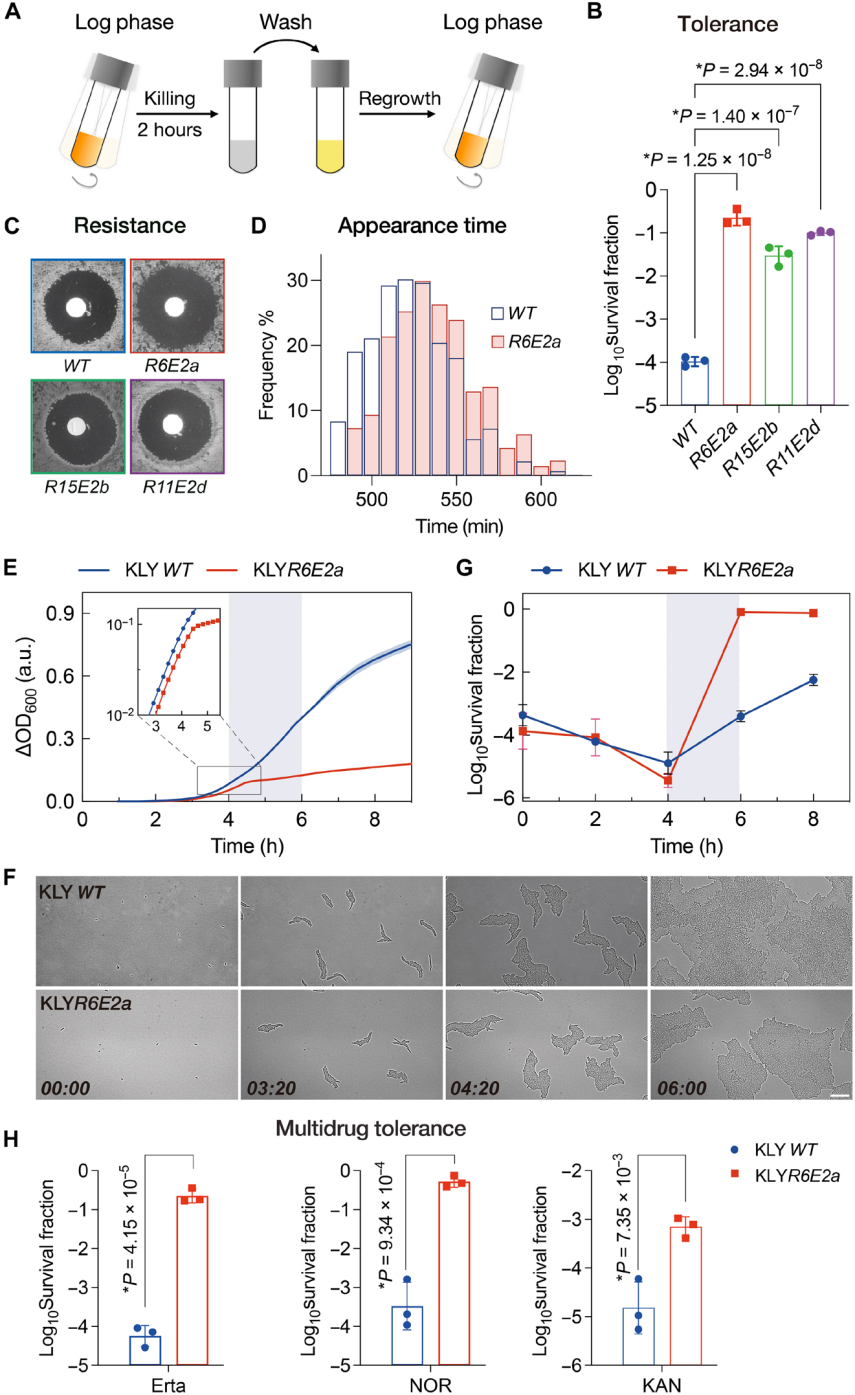
Premature growth arrest drives antibiotic persistence. (**A**) Cyclic antibiotic exposure: Log-phase cultures were treated with ertapenem (2 μg/ml) for 2 hours, washed to remove the drug, and resuspended and regrown in fresh LB to ∆OD_600_ ≈ 0.1 (see the shaded phase in fig. S1). (**B**) Survival of the ancestral and evolved strains after ertapenem (2 μg/ml) treatment for 3 hours. Data are presented as the means ± SD (*n* = 3). Clones *R6E2a*, *R15E2b*, and *R11E2d* were isolated from the 6th, 15th, and 11th rounds of evolution, respectively. (**C**) MIC test performed using disc diffusion antibiotic sensitivity testing with 1 μg of ertapenem. (**D**) Measurement of the lag-time distribution using the automated scanner system ScanLag. The histograms show the proportion of CFU detected at each time point. Mean lag times are 524.5 min [wild type (*WT*)] and 540.1 min (*R6E2*a). Sample sizes are *n* = 189 (*WT*) and 180 (*R6E2a*). (**E**) Growth curves of the evolved *R6E2a* and ancestral strains in LB. Cultures were grown in a 96-well plate, and OD_600_ was measured over time. Inset: The log scale reveals biphasic kinetics in the mutant. Shaded: growth arrest in KLY*R6E2*a. h, hours; a.u., arbitrary units. (**F**) Phase-contrast images from time-lapse microscopy of single cells from an exponential culture plated on an LB agar pad, showing the growth arrest in the *R6E2a* strain. Scale bar, 20 μm. Times are indicated in hours and minutes. (**G**) Persister fraction dynamics over the cell growth duration. Cultures were sampled at an interval of 2 hours for an antibiotic killing assay. Shaded: accelerated persister accumulation in KLY*R6E2a* at 4 to 6 hours of growth. (**H**) Survival fractions of the ancestral strain and the evolved KLY*R6E2* strain after 3 hours of treatment with ertapenem (Erta; 2 μg/ml), norfloxacin (NOR; 10 μg/ml), and kanamycin (KAN; 100 μg/ml). Data are presented as the means ± SD. *P* values were calculated using two-tailed *t* tests.

We observed that exponentially growing populations of wild-type *Escherichia coli* exhibited a marked increase in the fraction of drug-tolerant persisters (fig. S1A) (*32*). Parallel log-phase cultures of *E. coli* KLY, near the onset of persistence elevation (shaded area in fig. S1A), were periodically exposed to a high concentration of ertapenem [2 μg/ml, ~100-fold the minimum inhibitory concentration (MIC); fig. S1B and Fig. 1A]. Over several cycles, the killing efficiency of ertapenem declined by more than two orders of magnitude (Fig. 1B). Critically, the MICs of ertapenem for evolved clones remained indistinguishable from that for the ancestral strain, indicating that the enhanced survival was due to tolerance rather than resistance (Fig. 1C). Whole-genome sequencing revealed convergent *serS* mutations across independently evolved lineages (table S1), and these mutants displayed comparable exponential-phase growth defects (fig. S1C), prompting further investigation into growth arrest–associated persistence mechanisms.

The tolerant strain KLY*R6E2a*, established during the sixth treatment cycle, was selected for detailed characterization. While exhibiting a similar appearance-time distribution (Fig. 1D) and initial growth rate (Fig. 1E, inset) as the ancestor, the KLY*R6E2a* mutant underwent an abrupt growth arrest during the mid-exponential phase [change in optical density at 600 nm (∆OD_600_) ≈ 0.1; Fig. 1E]. Live imaging revealed continuous division in control KLY cells, whereas mutant cells exhibited biphasic growth dynamics, with an initial proliferation phase (0 to 4 hours) followed by a marked growth arrest (Fig. 1F, fig. S1D, and movie S1). This arrest in KLY*R6E2a* strongly correlated with enhanced persistence (Fig. 1, E and G), in line with ertapenem’s preferential killing of fast-growing cells (*33*). Moreover, the protective effect extended to norfloxacin and kanamycin exposure, indicating a multidrug tolerance phenotype (Fig. 1H). Overall, these mutants abruptly halted proliferation while maintaining viability, conferring tolerance to transient bactericidal antibiotic exposure.

### Serine exhaustion triggers growth arrest and dormancy

**Fig. 2.**
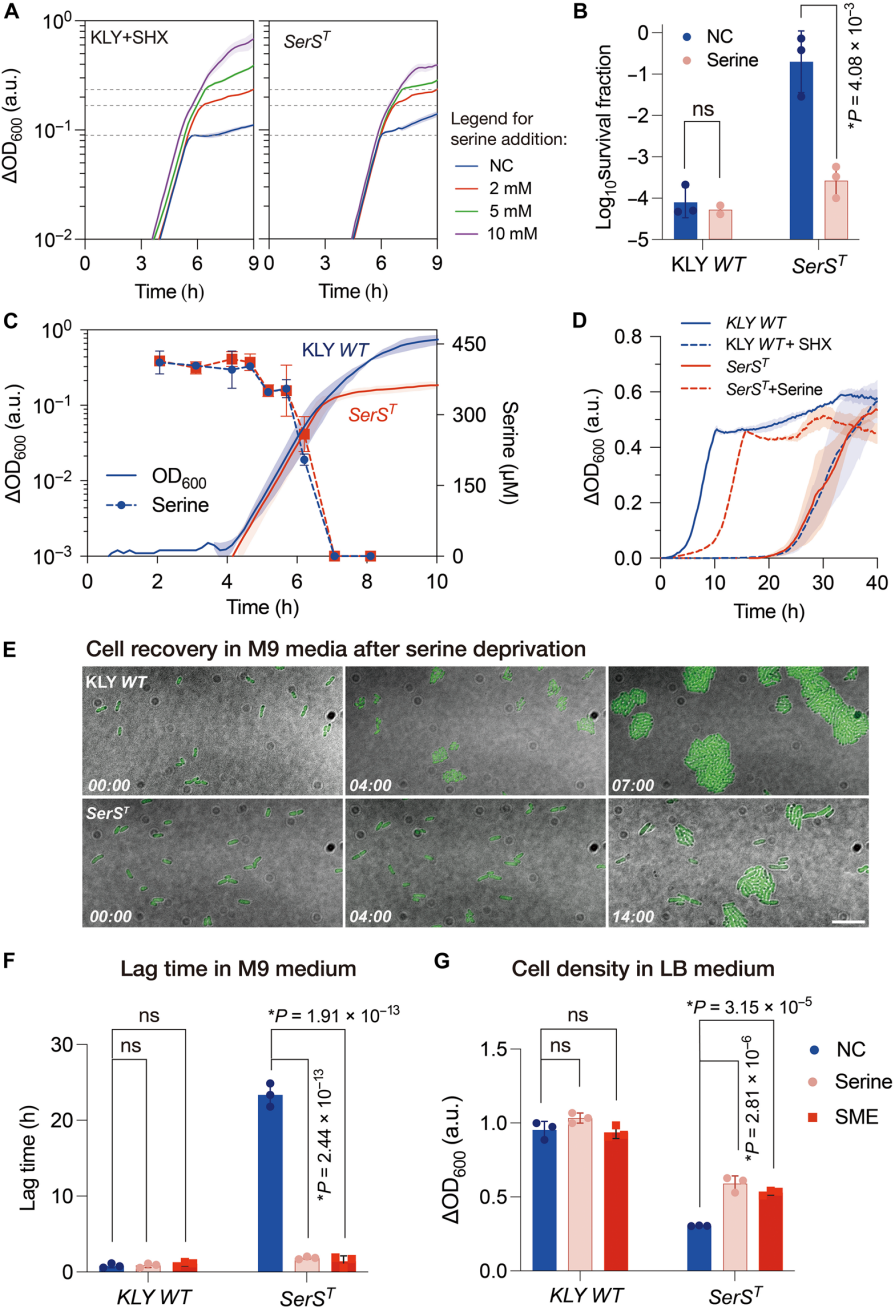
Serine and its analogs govern growth-arrested bacterial dormancy. (**A**) Growth curves of SHX-treated KLY and *SerS^T^* strains in LB medium supplemented with varying concentrations of serine. SHX, 1 mM. (**B**) Survival of the ancestral and evolved strains following ertapenem (2 μg/ml) treatment for 3 hours, with (serine) or without (NC) 2 mM serine supplementation in LB medium. Data are presented as the means ± SD of three independent experiments. ns, not significant. (**C**) Growth curves of the KLY and *SerS^T^* strains, along with the serine consumption profiles over the entire growth period. (**D**) Growth curves of the ancestor KLY and *SerS^T^* strains and of the SHX-treated KLY and serine-rescued *SerS^T^* strains. Cultures were initially grown in LB medium until ∆OD_600_ ≈ 0.1 and then transferred to M9 medium via a 1:1000 dilution. SHX, 1 mM; serine, 0.1 mM. (**E**) Time-lapse microscopy of single cells transferred from LB cultures to the M9 minimal medium, showing the extended lag time of the evolved strain. Time points are indicated in hours and minutes. Scale bar, 10 μm. (**F**) Serine and its analogs significantly reduced the lag time in M9 medium. Serine and SME were both used at 0.1 mM. (**G**) Serine and its analogs restored the serine deprivation–triggered growth arrest in LB medium. Serine and SME were both used at 5 mM. Data are presented as the means ± SD. *P* values were calculated using two-tailed *t* tests. h, hours.

Serine hydroxamate (SHX), a starvation-inducing serine analog and competitive inhibitor of seryl-tRNA synthetase (SerRS), has been shown to inhibit bacterial growth, a phenotype alleviated by serine addition (*34*–*38*). The *SerS^T^* (KLY*R6E2a*) mutant exhibited a growth-inhibitory phenotype closely resembling that of SHX-treated KLY cells, both of which were comparably rescued by increasing serine concentrations (Fig. 2A and fig. S2A). Mutant cultures were resensitized to antibiotic treatment following serine supplementation (Fig. 2B), and this growth arrest was specifically restored by serine, the cognate substrate of SerRS, rather than other amino acids (fig. S2B). These results suggest that persistence associated with growth arrest in the *SerS^T^* mutant arises from serine deficiency, consistent with previous findings that bacteria preferentially use serine over other amino acids during growth (*39*–*41*). Supporting this, liquid chromatography–mass spectrometry (LC-MS) analyses revealed a sharp depletion of extracellular serine during the exponential phase (fig. S2C). Upon serine exhaustion, the *SerS^T^* mutant rapidly entered growth arrest despite the availability of alternative nutrients, whereas the ancestral strain continued proliferating until 7 to 8 hours before exhibiting marked growth deceleration (Fig. 2C). Reproducing these growth patterns in minimal medium augmented with 20 amino acids (M9 + 20AA) established serine exhaustion as a critical factor driving the growth-arrested persistence in the *SerS^T^* strain (fig. S2, D and E).

Upon transfer to fresh M9 medium, the *SerS^T^* strain, preexposed to serine exhaustion in LB, exhibited a markedly prolonged lag phase compared to KLY, while subsequent growth rates were comparable (Fig. 2D), resembling typical “lag-dependent persistence” (*2*, *15*). This extended dormancy was absent when cells were transferred before serine exhaustion, indicating that serine availability governs the dormancy entry of the *SerS^T^* cells (fig. S3A). Time-lapse microscopy revealed that ancestral KLY cells resumed division within 4 hours, while a substantial fraction (~60%) of *SerS^T^* cells remained dormant even after 16 hours (Fig. 2E, fig. S3B, and movie S2). Serine supplementation shortened the lag time of *SerS^T^* in a concentration-dependent manner (Fig. 2D and fig. S3C). Notably, SHX-treated KLY cells displayed similarly prolonged lag dynamics, mirroring the dormancy observed in *SerS^T^* and revealing a previously unrecognized phenotypic switch induced by SHX beyond simple growth inhibition (Fig. 2D and fig. S3D) (*34*, *36*). Overall, these findings indicate that the *SerS^T^* mutant recapitulates the well-established persistence induced by SHX, with both growth arrest and extended dormancy triggered by serine exhaustion, suggesting a common underlying mechanism. Given that the wild-type KLY (*relA1* and *spoT1*) is a relaxed strain defective in (p)ppGpp synthesis (*42*, *43*), the growth-arrested dormancy in the *SerS^T^* or SHX-treated KLY strains points to a stringent response–independent mechanism (*34*, *44*, *45*). This conclusion is further supported by a similar growth arrest in the *E. coli* BW25113 *SerS^T^* strain, which harbors an intact stringent response system (fig. S3G) (*46*).

Although glycine and, to a limited extent, threonine can serve as metabolic substitutes for serine (*47*), neither alleviated the growth arrest observed in LB medium (fig. S2B). SHX-induced persistence is canonically attributed to the competitive inhibition of SerRS, impairing tRNA^Ser^ aminoacylation and disrupting protein synthesis (*48*, *49*). Notably, serine analogs, including serine methyl ester (SME), serinamide, and SHX, act as antimetabolites and competitive inhibitors of SerRS (*49*) yet paradoxically facilitated dormancy exit in *SerS^T^* cells grown in M9 medium (fig. S3, D to F, and Fig. 2F) and restored growth arrest in LB medium as effectively as serine (Fig. 2G). A possible explanation is that serine and its analogs exert regulatory effects on bacterial dormancy through interactions with the SerRS variant (SerRS^T^), rather than solely serving as metabolites or tRNA substrates.

### SerRS^T^ preferentially translocates into DEAD-box ATPase–marked condensates

Emerging evidence suggests that bacterial dormancy may involve protein condensates (*19*–*22*). In eukaryotic cells, adenosine 5′-triphosphate (ATP)–dependent helicases, RNA binding proteins, chaperonins, and aaRSs are conserved components of biomolecular condensates implicated in various biological processes, such as degenerative disease, circadian clock regulation, and cell fate decisions (*24*, *50*, *51*). Considering the involvement of SerRS^T^ in serine-dependent bacterial dormancy, we hypothesized that it might associate with biomolecular condensates upon serine deprivation, potentially contributing to antibiotic persistence.

**Fig. 3.**
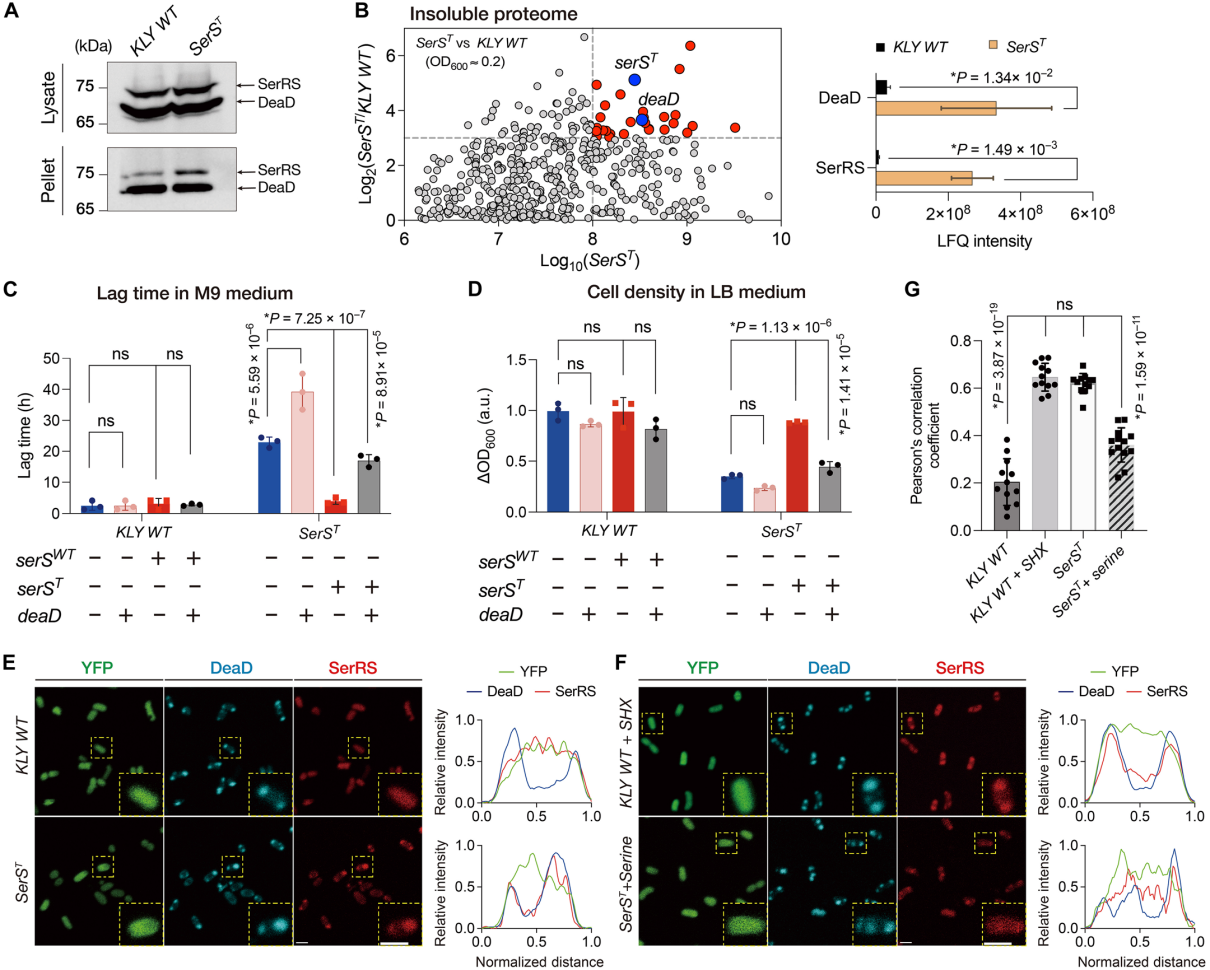
SerRS^T^ translocates into the DeaD-marked condensates upon serine deprivation. (**A**) Immunoblotting of endogenous SerRS-mCherry in the total lysate and insoluble pellet of the KLY *WT* and *SerS^T^* strains. His-tagged DeaD was expressed from a plasmid and induced with tetracycline (0.025 μg/ml). (**B**) MS analysis of insoluble proteome in *SerS^T^* versus KLY *WT*. Left panel: scatterplot showing the protein level changes. The *y* axis shows log_2_ fold changes of the *SerS^T^* strain over the KLY strain, and the *x* axis represents the log_10_ protein abundance of insoluble condensates in the mutant. Right panel: label-free quantitation (LFQ) intensity of SerRS and DeaD. Data are the means ± SD (*n* = 3). *P* values were calculated using two-tailed *t* tests. (**C** and **D**) Lag time in M9 (C) and cell density in LB (D) were modulated by the overexpression of DeaD and SerRS. Inducer concentrations were 1 mM for IPTG for DeaD expression under the *P_Lac1_* promoter and 0.05 μg/ml for tetracycline for the expression of *serS^WT^* or *serS^T^* under the *P_tet_* promoter. Data are the means ± SD*. P* values were calculated using two-tailed *t* tests. (**E**) Representative images of mCherry-tagged SerRS in cells expressing ECFP-tagged DeaD. (**F**) Representative images of mCherry-tagged SerRS in cells expressing ECFP-tagged DeaD with the addition of 10 mM SHX in KLY *WT* cells and 10 mM serine in the *SerS^T^* cells. Insets: higher magnification of yellow boxed areas. Line scans show the related intensity profiles of SerRS^WT^ with DeaD in ancestral cells and SerRS^T^ with DeaD in mutant cells of the magnified area. Cell lengths were normalized, and fluorescence was measured along a line across the cell long axis. Scale bars, 2 μm. (**G**) Pearson’s correlation coefficients quantifying DeaD-SerRS colocalization in KLY *WT* (*n* = 12), KLY *WT* + SHX (*n* = 12), *SerS^T^*(*n* = 12), and *SerS^T^* + serine (*n* = 13) cells. Data are the means ± SD. *P* values were calculated using two-tailed *t* tests.

To investigate the subcellular localization of SerRS, we inserted an mCherry tag into the endogenous *serS^wt^* or *serS^T^* loci in genomes using CRISPR-Cas9, enabling real-time tracking in living cells. Microscopy revealed that SerRS^T^, but not SerRS^wt^, preferentially localized to cell-pole granules during the early stationary phase (fig. S4A). This observation was further confirmed by coimmunoprecipitation, which showed that SerRS^T^ was markedly enriched in the insoluble condensate fraction compared with SerRS^wt^ (Fig. 3A). Quantitative mass spectrometry validated this enrichment and additionally revealed that the RNA-dependent DEAD-box adenosine triphosphatase (ATPase) DeaD (encoded by the *deaD* gene) was highly abundant in the insoluble pellets of the *SerS^T^* strain (Fig. 3B and fig. S4B). RNA-dependent DEAD-box ATPases have functioned as scaffolds for biomolecular condensates across diverse species (*52*, *53*). In both prokaryotic and eukaryotic cells, DEAD-box ATPases contribute to the dynamic assembly and turnover of membraneless organelles in response to internal and external signals (*52*–*55*). These findings suggest that the DeaD protein may participate in SerRS^T^-associated condensates and regulate bacterial dormancy and antibiotic persistence.

To test this hypothesis, we introduced a plasmid expressing DeaD into the *SerS^T^* strain, leading to a marked extension of the lag time (Fig. 3C). Induced expression of the mutated *serS^T^* gene alleviated the growth defect in LB medium and shortened the prolonged lag phase in M9 medium under finely tuned inducer concentrations, indicative of a dosage-sensitive mechanism rather than a canonical loss-of-function effect (Fig. 3, C and D, and fig. S5A). However, the growth-rescuing effect of *serS^T^* overexpression was abolished by the coexpression of the DeaD protein, suggesting that both DeaD and SerRS^T^ function as regulators of growth dynamics with opposing effects (Fig. 3, C and D, and fig. S5B). The highly concentration-dependent regulation of dormancy by proteins DeaD and SerRS exhibits notable mechanistic parallels with phase-separated biomolecular condensates (*56*). Knockout of the *deaD* gene in the *SerS^T^* tolerant strain minimally affected cell growth and lag time (fig. S4C), potentially due to functional redundancy with other DEAD-box ATPases, such as SrmB and RhlE (*57*).

Fluorescence microscopy of the KLY and *SerS^T^* strains coexpressing mCherry-tagged SerRS (SerRS^WT^-mCherry or SerRS^T^-mCherry) and enhanced cyan fluorescent protein (ECFP)–tagged DeaD showed that SerRS^T^ colocalized with DeaD-marked condensates upon growth arrest in the mutant, whereas SerRS^WT^ remained diffusely distributed in KLY cells (Fig. 3, E and G), a pattern also observed during the naturally occurring stationary phase (fig. S4D). Before growth stasis, both SerRS^WT^ and SerRS^T^ were uniformly distributed throughout the cytoplasm (fig. S4, D and E). Moreover, the DEAD-box helicases SrmB and RhlE colocalized with DeaD, suggesting that they may function redundantly with DeaD as modulators of condensates implicated in persistence regulation (fig. S4, F to I). Given that SHX treatment induced a growth defect similar to that of the *SerS^T^* strain, we next examined whether the SerRS^WT^ protein could translocate into DeaD-marked granules under these conditions. As hypothesized, SHX treatment triggered the compartmentalization of SerRS^WT^ into DeaD-marked condensates in KLY cells, recapitulating the phenotype observed in the *SerS^T^* mutant (Fig. 3, F and G). Consistent with previous findings, serine supplementation also reversed this sequestration, releasing a substantial fraction of SerRS^T^ from DeaD-marked granules (Fig. 3, F and G). Collectively, these findings support the idea that translocation of SerRS into DeaD-marked condensates upon serine depletion underlies growth-arrested persistence and bacterial dormancy, whether mediated genetically via the SerRS variant or environmentally via SHX treatment.

Building on these observations, we performed time-lapse microscopy to demonstrate that the SerRS^T^-DeaD condensates act as a reversible phenotypic switch. Transferring *SerS^T^* cells to the serine-limited condition triggered rapid formation of the SerRS^T^-DeaD condensates (fig. S6A), coinciding with growth arrest and enhanced survival under antibiotic stress (fig. S7). Conversely, reexposure to serine enabled the dormant persisters to resume proliferation, accompanied by gradual condensate dissolution, as evidenced by the progressive dispersal of SerRS^T^ foci over time (fig. S6B). Together, these results underscore serine availability as a key environmental cue that dynamically regulates condensate assembly, thereby establishing SerRS^T^-DeaD condensates as a reversible molecular switch governing the transitions between growth and dormancy.

### DeaD undergoes phase separation to sequester SerRS^T^ in condensates

**Fig. 4.**
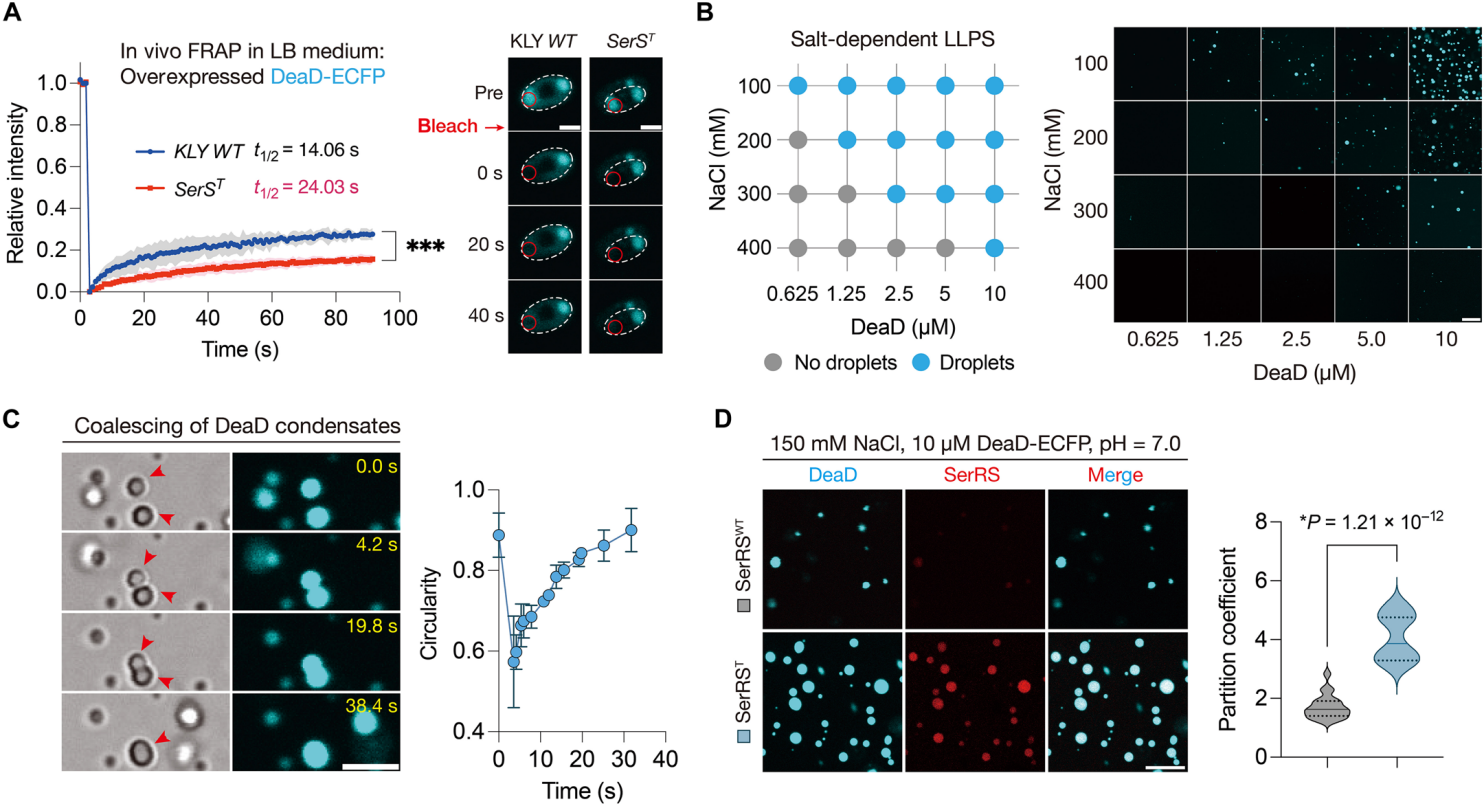
DeaD condensates selectively recruit the SerRS^T^ protein through LLPS. (**A**) FRAP analysis of DeaD granules in the ancestral KLY and *SerS^T^* mutant cells cultured in LB medium. Data are the means ± SD. ***P < 0.001 by a two-way analysis of variance (ANOVA) with Sidak’s multiple comparison test. Mobile fractions were 27% (KLY *WT*) and 16% (*SerS^T^*). Scale bars, 1 μm. (**B**) LLPS of purified recombinant DeaD at various amounts and NaCl concentrations. Left panel: summary of phase-separation behavior of protein DeaD; right panel: representative fluorescence microscopy images. Scale bar, 20 μm. (**C**) Coalescing DeaD condensates. Left panel: time-lapse imaging. Right panel: circularity of fusing condensates over time. Data are the means ± SD. *n* = 3 condensates. Scale bar, 5 μm. (**D**) SerRS^T^ partitioning by DeaD condensates in vitro. Left panel: microscopy images showing the enrichment of purified SerRS^T^ protein (red font and image, with a C-terminal mCherry tag) by partitioning to DeaD condensates (cyan font and image) in vitro. Right panel: quantification of 561-nm emission inside and outside DeaD condensates. Solid lines indicate the median. *P* values were calculated using two-tailed *t* tests; *n* = 20 condensates. Scale bar, 10 μm.

Given that liquid-liquid phase separation (LLPS) is a critical mechanism underlying biomolecular condensate formation in bacteria and eukaryotes (*20*, *58*–*61*), we investigated whether DeaD undergoes LLPS to form condensates that preferentially partition SerRS^T^ over SerRS^WT^. Fluorescence recovery after photobleaching (FRAP) measurements revealed that DeaD condensates in the *SerS^T^* strain showed significantly slower recovery kinetics and a higher immobile fraction, indicating that SerRS^T^ perturbs the dynamics of DeaD condensates in vivo (Fig. 4A). This effect was particularly pronounced in M9 medium, coinciding with the prolonged lag phase observed in mutant cells (fig. S8A). In vitro, DeaD protein underwent phase separation depending on protein and salt concentrations (Fig. 4B) (*52*). We then confirmed that DeaD condensates coalesce (Fig. 4C), and FRAP showed the recovery of fluorescence signal (fig. S8B), indicating their liquid-like material properties. We hypothesized that DeaD condensates function as scaffolds, whereas SerRS^T^ acts as a partitioning client molecule. Consistent with this, purified SerRS^T^ was significantly enriched in DeaD condensates, indicating that LLPS-mediated recruitment underlies its sequestration (Fig. 4D and fig. S8, C to E). To explore the basis of this selective recruitment, we analyzed the Gly^208^→Asp (G208D) substitution in the SerRS^T^ protein. This mutation introduces a negatively charged side chain that perturbs the local electrostatic balance without altering the overall protein structure (fig. S8F). Given that LLPS often involves electrostatic interactions between charged residues and termini (*62*), this charge alteration may enhance the propensity of SerRS^T^ to engage in such interactions, thereby promoting its preferential partitioning into DeaD-marked condensates via a charge-driven LLPS mechanism.

### SerRS sequestration into DeaD-marked condensates disrupts compartmentalized translation

**Fig. 5.**
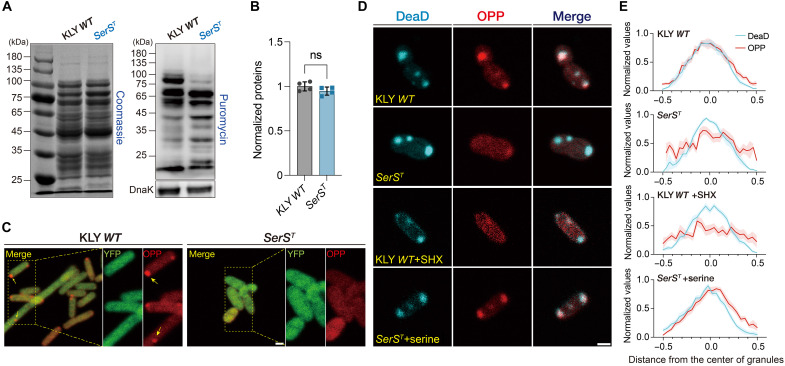
SerRS^T^ sequestration disrupts compartmentalized translation in DeaD-marked granules. (**A**) Western blot analysis showing newly synthesized polypeptides labeled with puromycin in KLY *WT* and *SerS^T^* cells upon serine exhaustion. Labeling time: 30 min. The total protein is shown as a reference for normalization. (**B**) Quantification of puromycin incorporation normalized to the total protein. Individual data points represent biological replicates, and data are represented as the means ± SD; the *P* value was calculated using an unpaired *t* test. (**C**) Microscopic images of the KLY *WT* and *SerS^T^* cells treated with OPP and stained with Alexa Fluor 594, illustrating active translation upon serine starvation. Yellow arrows pointed to active translation sites. Scale bar, 1 μm. (**D**) Colocalization of nascent peptides and DeaD-marked condensates in KLY *WT* and *SerS^T^* cells expressing ECFP-tagged DeaD. Altered colocalization patterns were observed in SHX-treated KLY *WT* cells and serine-restored *SerS^T^* cells. SHX, 1 mM; serine, 2 mM. Scale bar, 1 μm. (**E**) Signal intensity profiles of OPP-labeled nascent polypeptides (red), centered at DeaD-marked condensates (cyan). Shaded areas around lines indicate SEMs (*n* = 10, 10, 11, and 13).

Canonical models propose that mutations in aaRSs drive persistence by impairing tRNA charging, thereby inhibiting protein translation and cell proliferation (*26*–*29*). To test this, we measured the aminoacylation activity of the SerRS^T^ protein. In vitro assays confirmed reduced catalytic efficiency, consistent with decreased tRNA^Ser^ charging levels observed in the *SerS^T^* mutant strain (fig. S9, A and B). To evaluate the impact on global protein synthesis, we next used the SUnSET (surface sensing of translation) assay, in which puromycin incorporates into nascent polypeptides as an aminoacyl-tRNA mimic (*63*). Unexpectedly, despite reduced Ser-tRNA levels, the overall translation efficiency was negligibly affected. Nevertheless, the *SerS^T^* strain exhibited markedly altered translation patterns compared to KLY (Fig. 5, A and B). These findings indicate that sufficient Ser-tRNA remains available for protein synthesis, suggesting that growth arrest in the *SerS^T^* mutant is not due to global translation inhibition. Notably, low Ser-tRNA levels are a common physiological feature in *E. coli*, particularly during rapid growth, where charging levels can drop below 10% as part of normal cellular adaptation (*64*, *65*).

In eukaryotes, biomolecular granules can regulate translation by recruiting components of the translational machinery or modulating mRNA dynamics to orchestrate cell-state transitions (*50*, *51*, *66*). Inspired by these findings, we speculated that the altered translation patterns in the *SerS^T^* mutant reflect a spatial reorganization of protein synthesis involving DeaD-marked condensates. To investigate this, we used *o*-propargyl-puromycin (OPP) coupled with click-reaction labeling to visualize newly synthesized polypeptides (*63*). In ancestral KLY cells, translation hotspots were distinctly localized at the cell poles or mid/quarter-cell positions, whereas these sites were largely absent in *SerS^T^* cells (Fig. 5C). The spatial proximity between DeaD-marked condensates and translation hotspots in KLY suggests that these granules may serve as hubs for localized protein synthesis, a function disrupted by SerRS^T^ sequestration. Supporting this idea, DeaD-marked condensates in KLY exhibited strong colocalization with sites of active translation (Fig. 5, D and E, top); by contrast, these translation hotspots were largely lost from DeaD granules in *SerS^T^* cells (Fig. 5, D and E, second row). Both SerRS^T^ and SHX-treated SerRS^WT^ partitioned into DeaD condensates, suggesting a shared mechanism for repressing condensate-associated translation. In wild-type KLY cells, SHX treatment recapitulated the growth defect and the altered translation patterns seen in the *SerS^T^* mutant (fig. S9, C and D), substantially reducing protein synthesis within DeaD-marked condensates (Fig. 5, D and E, third row). Conversely, serine supplementation restored translation hotspots in *SerS^T^* cells, indicating that these spatial translation defects are reversible upon SerRS release, which is triggered by serine availability (Fig. 5, D and E, fourth row).

Together, these results indicate that DeaD-marked condensates closely associate with sites of localized translation and that aberrant SerRS sequestration disrupts this process. The proteins synthesized within these granules could include factors directly involved in growth regulation, providing a potential mechanistic link between localized translation modulation and cell proliferation. Notably, a similar reduction in translational activity within DeaD condensates was consistently observed across other evolved lineages and stationary-phase cells and in the *E. coli* BW25113 strain with a functional stringent response system (fig. S9E) (*46*), highlighting the generality of this mechanism in bacterial dormancy.

### SerRS^T^ partitioning remodels translational components within DeaD-marked condensates

**Fig. 6.**
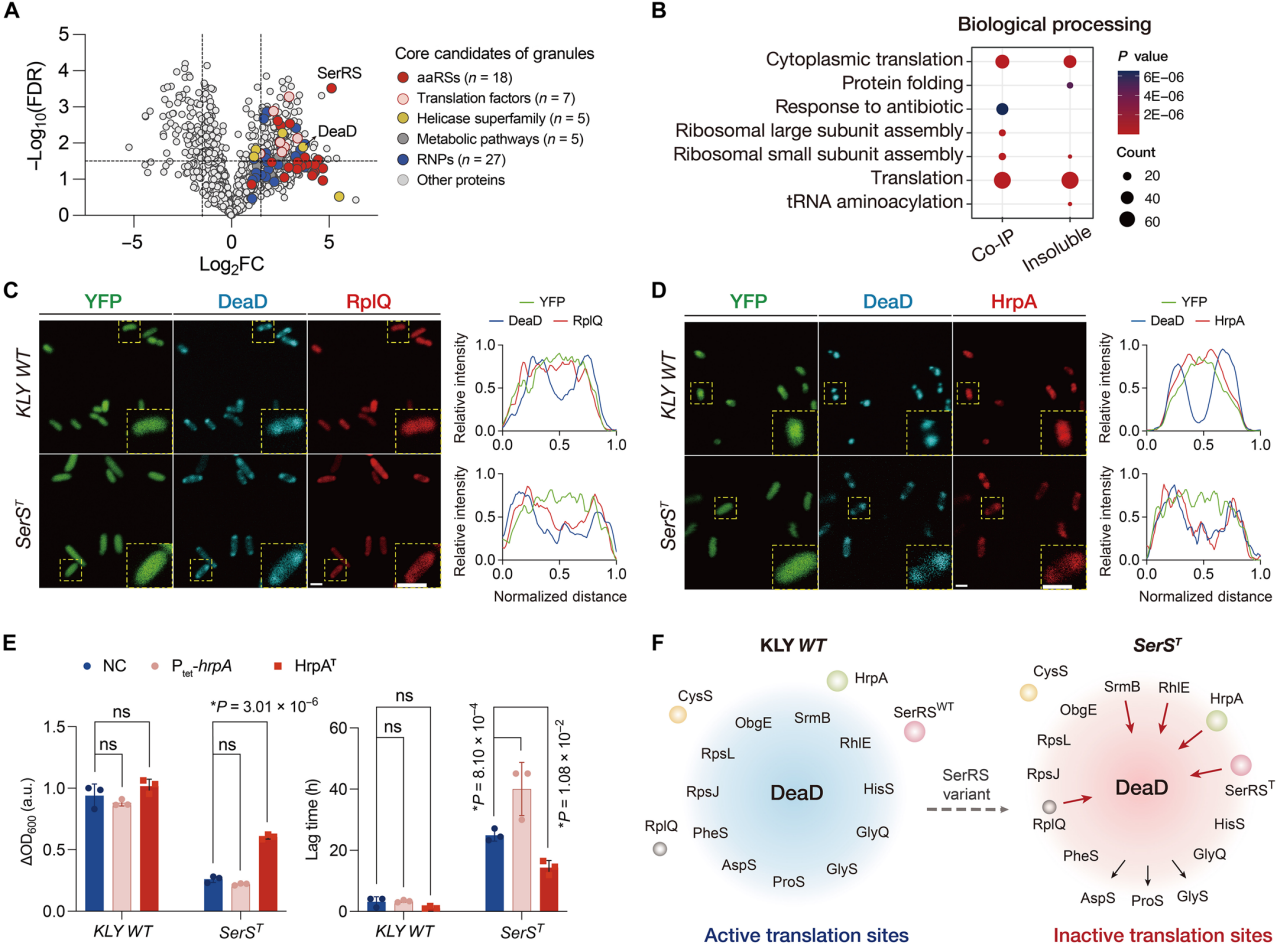
SerRS^T^ partitioning remodels the translational network within DeaD-marked condensates. (**A**) Volcano plots displaying the MS analysis of insoluble fractions from the *SerS^T^* strain relative to the KLY *WT* strain. Statistical significance was assessed using a *t* test with the false discovery rate (FDR) correction–adjusted *P* < 0.02. FC, fold change; RNPs, ribonucleoproteins. (**B**) Gene ontology analysis of immunoprecipitated proteins from the SerRS^T^ versus SerRS^WT^ interactomes and components enriched in the insoluble pellets of mutated versus wild-type cells. Co-IP, coimmunoprecipitation. (**C** and **D**) Representative images of mCherry-tagged ribonucleoprotein RplQ (C) or HrpA (D) in ancestral and *SerS^T^* cells expressing ECFP-tagged DeaD. Inset: higher magnification of the yellow boxed area. Scale bar, 1 μm. Line scans show the intensity profiles of DeaD colocalized with mCherry-tagged candidates of the magnified area. Pearson’s correlation coefficients quantifying colocalization of DeaD-RplQ and DeaD-HrpA are shown in fig. S10 (F and G). (**E**) Effect of HrpA mutants on the growth defect. The expression plasmid of HrpA was induced by tetracycline (0.05 μg/ml), and the truncated mutation of *hrpA* was generated in the genomes of both *SerS^T^* and ancestral strains via the CRISPR-Cas9 system. Data are the means ± SD; *P* values were calculated using two-tailed *t* tests. (**F**) Schematic depicting the altered translocation of translational components within DeaD-marked condensates. See also fig. S11.

LLPS-driven biomolecular condensates, such as P-bodies and stress granules, dynamically adjust their protein composition in response to environmental cues or genetic mutations (*50*, *67*). To investigate how SerRS^T^ sequestration disrupts the translational function of DeaD-marked condensates, we performed proteomic analysis of the insoluble fraction from the KLY and *SerS^T^* strains, identifying ~500 candidates with altered translocation (Fig. 6A). Gene functional classification revealed that many of these proteins are core components of the translational machinery, including aaRSs, ribonucleoprotein complexes, translation factors, and ATP-dependent helicases (Fig. 6A). These findings were further validated in the SerRS-associated interactome using coimmunoprecipitation, a method that preserves the integrity of the protein complex (fig. S10, A to C). Gene ontology analysis of SerRS-immunoprecipitated proteins and insoluble condensate fractions highlighted the strong enrichment of translational processes and ribosomal subunit assembly in *SerS^T^* relative to the ancestral KLY strain, suggesting a pronounced reorganization of translation components within DeaD-marked condensates (Fig. 6B).

Building on the proteomic analysis, we next examined a subset of translocated candidates to assess their functional effects on *SerS^T^* growth. Notably, overexpression of certain ribosomal subunits, particularly L17 RplQ (encoded by *rplQ*), significantly rescued the growth defect in the *SerS^T^* strain (fig. S10D). Fluorescence microscopy revealed enhanced colocalization of RplQ with DeaD-marked condensates in *SerS^T^* cells compared to wild-type cells (Fig. 6C and fig. S10F). To further identify proteins that modulate the SerRS^T^-associated growth defect, we used a reverse selection strategy using cyclic batch culturing to enrich mutants with a growth advantage over the *SerS^T^* strain (fig. S10E). In addition to *serS^T^*, these growth-restored mutants could harbor a second mutation that potentially counteracts the SerRS^T^ function. Among these, a truncated version of HrpA (designated HrpA^T^, c.2684delA) was identified, whose wild-type allele was also enriched in the SerRS^T^ interactome (fig. S10C). *E. coli* HrpA, an ATP-dependent RNA helicase of the DEAH/RHA family, has been implicated in modulating antibiotic susceptibility, possibly through effects on RNA dynamics and bacterial stress responses (*68*–*70*). Fluorescence imaging showed that mCherry-tagged HrpA colocalized with DeaD-marked condensates in *SerS^T^* cells, whereas it displayed a diffuse cytoplasmic distribution in wild-type cells (Fig. 6D and fig. S10G). Moreover, overexpression of HrpA exacerbated the growth defect in *SerS^T^* cells, whereas this phenotype was partially restored in the *SerS^T^hrpA^T^* mutant (Fig. 6E). The opposite effects of RplQ and HrpA likely reflect their distinct functional contributions within DeaD-marked condensates, illustrating that the abundance of certain condensate-resident proteins can modulate condensate activity. We further confirmed interactions between DeaD and representative aaRSs in both mutant and ancestral strains. In *SerS^T^* cells, HisS, GlyQ, and PheS maintained or increased their interactions with DeaD-marked condensates, whereas GlyS, AspS, and ProS exhibited weaker interactions (Fig. 6F and fig. S11). Notably, CysS was consistently excluded from DeaD-driven granules. Overall, these findings suggest that SerRS^T^-driven alterations in condensate components could critically affect translational activity, highlighting condensates as regulatory hubs controlling bacterial dormancy and antibiotic persistence.

## DISCUSSION

We tracked the evolution of exponential-phase *E. coli* populations under cyclic antibiotic treatment and isolated a growth-arrested persistence that initiates dormancy before the onset of the stationary phase. This persistence is mediated by convergent mutations in the *serS* gene, akin to the inhibitory response seen in SHX-treated cells. Our results establish serine exhaustion as a critical driver of antibiotic persistence and bacterial dormancy, triggering the translocation of SerRS into DeaD-marked condensates. This translocation perturbs the interactome and disrupts compartmentalized translation, indicating a common persistence mechanism triggered by either the SerRS variant or SHX treatment.

Serine serves as a multifunctional metabolite. It contributes to protein and phospholipid synthesis and fuels the folate-mediated one-carbon cycle that supports nucleotide synthesis, methylation reactions, and antioxidant defense (*71*–*73*). The phenomenon of sharp serine consumption during the exponential phase has been well documented for decades and may indicate nutrient status, allowing bacteria to adjust their population size (*39*, *41*). In natural environments, as nutrient availability fluctuates between periods of feast and famine, bacteria likely integrate various environmental cues to establish the transition between proliferation and dormancy, such as the duration of dormancy and the timing of division. In eukaryotes, aaRSs can form multisynthetase complexes, which are increasingly recognized as mediators of cell signaling for extratranslational functions (*74*, *75*). Since the identification of high-persistence mutants, such as HipA7 and *MetG^T^*, the mechanisms by which aaRS-related pathways regulate dormancy remain inadequately understood (*2*, *28*). Our results suggest a potential general mechanism whereby aaRSs sense the levels of their cognate amino acids and partition into LLPS-mediated biomolecular condensates, thereby regulating cell fate in prokaryotes.

**Fig. 7.**
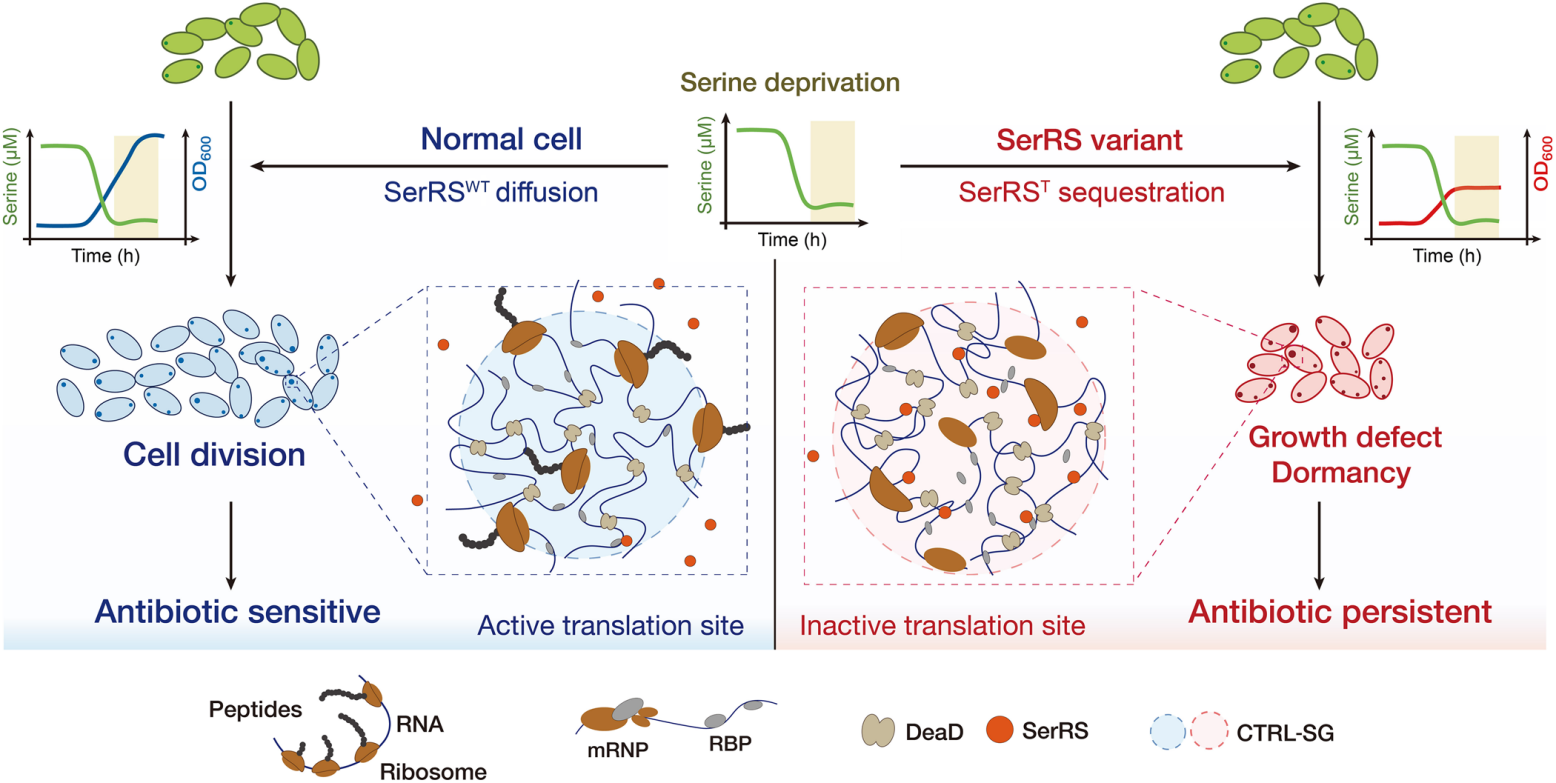
SerRS sequestration into CTRL-SGs modulates compartmentalized translation and antibiotic persistence. Upon serine deprivation, SerRS^T^ translocates into DEAD-box ATPase–marked condensates, where its sequestration disrupts compartmentalized translation and coordinates bacterial entry into dormancy, thereby promoting antibiotic persistence. These phase-separated condensates function as a reversible phenotypic switch, termed the CTRL-SG. h, hours.

Biomolecular condensates are membraneless intracellular assemblies that are tightly regulated in response to environmental signals. In eukaryotes, DEAD-box ATPases have emerged as conserved and redundant regulators of RNA-containing condensates, which in turn modulate stress responses and cell fate (*53*, *67*, *76*). Our findings suggest that analogous DeaD-marked granules may evolve in unicellular prokaryotes, enabling adaptive transitions from proliferation to dormancy in response to nutrient fluctuations. Notably, we observed that these condensates act as translation hubs, consistent with similar findings in eukaryotic cells, and may use comparable modes of translation regulation (*51*, *66*, *77*, *78*). Specifically, SerRS^T^ expression exhibited a biphasic response: Moderate levels alleviated the growth defect in the *SerS^T^* strain, whereas excessive expression reinstated growth inhibition (Fig. 3, C and D, and fig. S5A), closely resembling the concentration-dependent, bidirectional regulation of condensates in eukaryotes (*78*). Moreover, translational factors identified within DeaD-marked condensates correspond to conserved components of well-characterized granules (*24*, *55*, *79*). Serine deprivation triggers pronounced translocation of SerRS^T^ into DeaD condensates, forming reversible SerRS^T^-DeaD structures that disrupt localized translation and promote growth arrest, thereby facilitating entry into dormancy. Conversely, serine replenishment induces condensate dissolution, restoring compartmentalized translation and enabling persister resuscitation, establishing the SerRS^T^-DeaD condensates as a reversible phenotypic switch. We term these structures as the “compartmentalized translation-regulating dormancy switch granule” (CTRL-SG) in bacteria (Fig. 7).

Notably, our findings establish a strong correlation between serine exhaustion and SerRS recruitment into DeaD-marked granules. However, the precise determinants of this partitioning remain to be fully elucidated. Multiple factors may contribute, including direct SerRS-DeaD interactions, altered aminoacylation status, or RNA-mediated scaffolding, all of which could modulate the dynamics and composition of DeaD condensates. Future efforts using targeted mutants that either prevent SerRS recruitment or disrupt condensate integrity will help determine whether condensate-mediated SerRS partitioning is strictly required for persistence. Moreover, establishing a direct causal link between localized translation suppression and growth arrest remains a major challenge. Identifying RNAs and proteins selectively retained or translated within DeaD-marked condensates will be key to defining which growth-critical proteins are affected by condensate remodeling. These efforts are expected to provide mechanistic insight into how condensates control bacterial dormancy entry and may ultimately enable in vitro reconstitution assays to test how SerRS and DeaD levels modulate translation within granules. Beyond translation control, these condensates may also participate in RNA posttranscriptional processing, such as RNA editing, degradation, and tRNA modification (*55*, *80*). For example, HrpA, which processes the *daa* operon mRNA in *E. coli*, shares notable sequence similarity with yeast RNA helicases (PRP2, PRP16, and PRP22) involved in mRNA splicing (*68*, *69*, *81*). These associations suggest that CTRL-SGs may have evolved in prokaryotes to selectively modulate gene expression and confer resilience to diverse stresses, a function that remains to be fully explored.

Cell-to-cell heterogeneity is generally attributed to intrinsic gene expression noise (*82*, *83*), in which condensate formation has been suggested as a potential mechanism to reduce cytoplasmic noise (*84*) or, conversely, to amplify the cell-cell variability of condensate-regulated cellular physiology properties (*85*). SHX-induced perturbations were predicted to disrupt the cellular network, shifting its dynamics from biological adaptation toward the generic behavior of a large random network, rather than a specific genetic pathway. Inspired by this hypothesis, the analogy of serine starvation-triggered persistence mediated by SerRS^T^ mutation and SHX treatment prompted us to explore a similarly dysregulated network. This network may underlie the extensive heterogeneity in the physiology of bacterial dormancy, in line with the highly heterogeneous formation and regulation of phase-separated biomolecular condensates.

In conclusion, the identification of condensate-regulated compartmentalized translation in prokaryotes suggests that LLPS-driven, membraneless granules represent an evolutionarily conserved phenomenon. These structures provide a versatile model for investigating the mechanisms and evolution of stress responses and cell fate decisions. This insight may also inform potential strategies for targeting dormancy regulation and therapeutic interventions against persistence-associated infections.

## MATERIALS AND METHODS

### Strains and cell culture

The ancestral strain (KLY) was conducted by P1 transduction of the yfp-Cam cassette from M22 into *E. coli* KL16, as previously described (*2*). All experiments were conducted at 37°C in LB (Sigma-Aldrich) medium, shaking at 220 rpm, unless otherwise specified. Bacterial cultures were grown to the exponential phase and stored in aliquots with 15% glycerol at −80°C. Cell growth in M9 minimal medium [Sangon M9 salts, supplemented with 0.2% glucose as the carbon source, 0.1 mM CaCl_2_, 1 mM MgSO_4_, and thiamine·HCl (1 mg/liter)] was initiated by first growing the cultures in LB medium until ∆OD_600_ ≈ 0.1 (following serine starvation), after which they were transferred to M9 medium via a 1:1000 dilution. The cultures were then incubated at 37°C with shaking at 220 rpm.

### Construct

The expression plasmids of SerRS^WT^-mCherry and SerRS^T^-mCherry were constructed as follows. DNA fragments encoding SerRS^wt^ and SerRS^T^ were polymerase chain reaction (PCR)–amplified from the KLY *wt* and *SerS^T^* genomes, respectively, and inserted into a p15A-Ptet-Kan^R^ backbone using the ClonExpress II One Step Cloning Kit (Vazyme, C112-01). To generate DeaD expression plasmids, the DNA fragment encoding DeaD was PCR-amplified from the KLY *WT* genome, fused to an ECFP tag, and assembled into a pET28a backbone (Amp^R^), with the expression driven by a *P_lac1_* promoter induced by isopropyl β-d-thiogalactopyranoside (IPTG). His-tagged DeaD was assembled into a p15A-Ptet-Kan^R^ backbone. Other DEAD RNA-dependent ATPases, including SrmB, RhlE, and HrpA, were cloned from the KLY genome and assembled into the p15A-Ptet-Kan^R^ backbone. DNA fragments encoding ribonucleoproteins (RNPs) were PCR-amplified from the KLY *WT* genome, fused to mCherry, and inserted into a pET28a backbone (Kan^R^). The aaRS genes (GlyQ, GlyS, CysS, PheS, ProS, AspS, and HisS) were cloned from the KLY *WT* genome, fused to mCherry, and then assembled into the p15A-Ptet-Kan^R^ backbone. All constructs were confirmed by sequencing and are listed in table S2.

### Cyclic evolution design

The cyclic experimental design included three steps. First, 50-μl aliquots [~10^7^ colony-forming units (CFU)/ml] were inoculated into 50 ml of LB and cultured until the optical density at 600 nm (OD_600_) reached ~0.2 (∆OD_600_ ≈ 0.1). Second, cultures were exposed to ertapenem (2 μg/ml; ~100× MIC; Shanghai Yuanye Bio-Technology Co., Ltd.) for 2 hours. Last, the antibiotic-containing supernatants were removed by centrifugation (6000 rpm, 6 min), and cell pellets were washed twice with 1 ml of 0.9% NaCl, resuspended in 50 ml of fresh LB, and regrown to the exponential phase (∆OD_600_ ≈ 0.1). MIC values were determined by serial twofold dilutions in LB with shaking at 37°C for 24 hours and defined as the lowest concentration of antibiotic that inhibited visible growth.

### Sampling

Unless otherwise specified, LB-cultured cells used in this study were collected at an ∆OD_600_ of ~0.1 following serine starvation. At this point, the *SerS^T^* tolerant strain exhibited an abrupt growth arrest, while the KLY strain continued to divide.

### ScanLag

A sample of the culture was serially diluted and plated onto solid LB agar. These plates were then incubated in a ScanLag system (*86*) at 37°C, an array of office scanners that automatically captures images every 20 min to monitor the appearance of thousands of colonies. An automated image analysis tool processes the images to derive the distribution of colony appearance times.

### Killing assay

Bacterial cultures were grown in LB to ∆OD_600_ ≈ 0.1, supplemented with ertapenem (2 μg/ml), and incubated for 3 hours at 37°C shaking at 250 rpm. CFU were determined by plating before and after antibiotic treatment. All assays were performed in triplicate.

### Whole-genome sequencing

Strains were grown overnight in LB medium, and genomic DNA was extracted using the TIANamp Bacteria DNA kit (DP302). DNA samples from each strain were sent to Beijing Saimo Baihe Biotechnology Co., Ltd., for Illumina sequencing. Genomic analysis was performed using Geneious Prime to detect single-nucleotide polymorphisms and new junctions. Each reported single-nucleotide polymorphism was confirmed by PCR amplification and sequencing.

### Confocal microscopy

#### Time-lapse microscopy for recording bacterial growth

Cells in an exponential phase were collected, washed three times with LB medium, and imaged on a gel pad containing 1% agarose. The LB medium gel pad was prepared as a gel island at the center of a confocal dish (Cellvis, D35-20-1.5-N). Cells were monitored under differential interference contrast (Nikon Ti2-E) microscopy at 37°C using a live-cell system. To observe bacterial resuscitation under the minimal M9 gel pad, LB-cultured cells were collected, washed three times with M9 minimal medium, and placed under a fresh M9 gel pad, as described above. The cells were then imaged under bright-field and fluorescence illumination (Nikon AXR NSPARC) at 37°C.

#### Microscopy imaging

Images were captured using confocal imaging (Nikon AXR NSPARC) with a 100× oil objective. ECFP was excited at 445 nm, YFP (yellow fluorescent protein) at 488 nm, mCherry/Alexa Fluor 594 at 561 nm, and Alexa Fluor 594 at 594 nm. The fluorescence signals were collected using the following filter sets: 474/20 nm for ECFP, 540/20 nm for YFP, and 618/20 nm for mCherry/Alexa Fluor 594. Colocalization analysis was performed using Fiji (Colocalization Finder). A square region containing KLY *WT* or *SerS^T^* cells with condensates was selected as the region of interest (ROI). Pearson’s correlation coefficient between two channels within the ROI was calculated to quantify colocalization. For line-scan analysis, a straight line was drawn across the enlarged image of the ROI, and the intensity profile was measured using the Plot Profile tool in Fiji.

#### Translation visualization

Cells were incubated with OPP using the Click-iT Plus OPP Alexa Fluor 594 Protein Synthesis Assay Kit (Invitrogen) (*63*) according to the manufacturer’s guidelines. Briefly, cells we incubated with 20 μM OPP for 30 min at 37°C with shaking. Cells were then fixed with 4.0% formaldehyde and permeabilized with 0.1% Triton X-100 for 15 min at room temperature. Fluorescence labeling was performed for 30 min with an Alexa Fluor 594 reaction cocktail at room temperature.

### Detecting amino acid concentrations

Extracellular and intracellular amino acid concentrations were quantified by LC-MS. Metabolites were sampled at regular intervals during the testing phase and extracted using 80% methanol. The extracts were directly analyzed without drying. Metabolite quantification was performed using a 4500+ QTrap mass spectrometer (AB SCIEX, US) and an ACQUITY UPLC H-Class system (Waters, US). Chromatographic separation was achieved using an ACQUITY UPLC BEH Amide column (2.1 by 100 mm, 1.7 μm; Waters). Mobile phase A contains HPLC (high-performance liquid chromatography)–grade acetonitrile and 0.1% formic acid, while mobile phase B is water and 0.1% formic acid. Data were collected in positive mode using the multiple reaction monitoring mode with an electrospray ionization source. The gas pressures for the atomizer (gas 1), heater (gas 2), and curtain were set to 55, 55, and 30 psi, respectively. The ion spray voltage for the positive ion mode is 5500 V. The optimal probe temperature was determined to be 500°C, and the column oven temperature was set at 40°C. The AB SCIEX 1.6.3 analyzer (AB SCIEX, US) was used for metabolite identification and peak integration.

### Insoluble protein isolation and SDS-PAGE

Insoluble proteins were isolated from bacterial cultures following a modified method from Tomoyasu *et al.* (*87*) and Pu *et al.* (*19*). Briefly, 20 ml of bacterial culture (OD_600_ = 0.2) was rapidly cooled on ice. Cells were collected by centrifugation at 6000*g* for 10 min at 4°C. The pellet was resuspended in 40 μl of buffer A [10 mM potassium phosphate buffer, pH 6.5, 1 mM EDTA, 20% (w/v) sucrose, and lysozyme (1 mg/ml)] and incubated on ice for 30 min. The cell lysate was mixed with 360 μl of buffer B (10 mM potassium phosphate buffer, pH 6.5, and 1 mM EDTA) and subjected to sonication on ice. Membrane proteins were dissolved by resuspending the pellet in 400 μl of buffer C (buffer B with 2% NP-40). The insoluble proteins were collected by centrifugation at 15,000*g* for 30 min at 4°C. NP-40–insoluble pellets were washed twice with 400 μl of buffer B and resuspended in 50 μl of buffer B by brief sonication. Proteins were separated by 10% SDS–polyacrylamide gel electrophoresis (SDS-PAGE) and stained with Coomassie Brilliant Blue.

### Western blots

Protein concentrations were determined using a bicinchoninic acid protein assay kit (Beyotime, P0012). Proteins were separated on SDS-PAGE and transferred to a polyvinylidene difluoride membrane. The membranes were blocked in phosphate-buffered saline (PBS) containing 0.5% milk and 0.5% Tween 20 for 1 hour at room temperature. Primary antibodies were incubated overnight in PBS with Tween 20 containing 4% bovine serum albumin, 1% Tween 20, and 0.05% NaN_3_ and then incubated with the appropriate horseradish peroxidase–conjugated secondary goat antibodies (Beyotime, A0350) for 2 hours at room temperature, followed by extensive washing. Blots were detected using a GE western blotting substrate, and acquired images were analyzed with ImageJ software. Primary antibodies used in this study were mCherry (Cell Signaling, 43590), His-tag antibody (Sangon, D191001), anti-DnaK antibody (Abcam, ab80161), and anti-puromycin (AbClonal, A23031).

### Coimmunoprecipitation

Coimmunoprecipitation was performed using KLY wild-type and *SerS^T^* strains harboring an mCherry tag fused to the C terminus of SerRS protein. Cultures were grown to OD_600_ ≈ 0.2 (upon serine depletion) and lysed using lysis buffer (50 mM NaCl and 20 mM tris-HCl, pH 7.4) containing a protease inhibitor cocktail (Beyotime, P1005), Superase IN (1 U/ml; Invitrogen, AM2694), and ribonuclease-free deoxyribonuclease I (10 U/ml; Thermo Fisher Scientific, EN0521). Immunoprecipitation was performed with Anti-mCherry Nanobody Immunomagnetic Beads (Elabscience, EAIP-005MN). The precipitates were subjected to quantitative mass spectrometry to analyze the interacted protein candidates.

### Mass spectrometry

The SDS-PAGE gel was reduced with 5 mM dithiothreitol and alkylated with 11 mM iodoacetamide, followed by in-gel digestion with sequencing grade modified trypsin at 37°C overnight. Peptides were extracted twice with 0.1% trifluoroacetic acid in a 50% acetonitrile aqueous solution for 30 min and centrifuged in a SpeedVac to concentrate the extracts. The digestion products were separated via a 120-min gradient elution at a flow rate 0.300 μl/min on a Thermo Fisher Scientific UltiMate 3000 HPLC system, directly interfaced with the Thermo Orbitrap Fusion Tribrid mass spectrometer. The analytical column was a C18 fritless column (75 μm by 15 cm, Acclaim PepMap nanoViper RSLC Thermo). Mobile phase A consisted of 0.1% formic acid, and mobile phase B consisted of 80% acetonitrile and 0.1% formic acid. The Orbitrap Fusion was operated in data-dependent acquisition mode using Xcalibur 4.1 software, performing a full-scan mass spectrum (350 to 1550 mass/charge ratio, 120,000 resolution) followed by top-speed tandem MS scans in the Orbitrap. The tandem MS spectra were searched against the *E. coli* K-12 database using Proteome Discoverer (version PD3.1, Thermo Fisher Scientific, US).

### CRISPR-Cas9 genome editing

Genome editing of *serS^WT^*, *serS^T^*, *deaD*, and *hrpA* genes was performed via CRISPR-Cas9–mediated genome editing (*88*). Briefly, the plasmid Kan^R^ p15A-P_BAD_-Cas9-P_T5_-Redγβα (plasmid no. 1) was respectively transferred into the *E. coli* KLY *WT* and *SerS^T^* strains to obtain the corresponding transformants. Temperature-sensitive Amp^R^ plasmids were constructed to express specific sgRNA (single guide RNA) and generate specific donor DNA; these plasmids were collectively named pSC101-P_BAD_-sgRNA-Donor (plasmid no. 2). Plasmid no. 2 was introduced into competent cells (containing plasmid no. 1) and resuscitated for 45 min. Then, cells were plated on LB agar containing kanamycin and ampicillin and cultured overnight at 30°C. Following primary selection, cultures were inoculated into LB broth (containing kanamycin and ampicillin) and cultured at 30°C for 2 hours. IPTG was added, and the mixture was cultured for 1 hour at 30°C; l-arabinose was then added and incubation continued at 30°C for 3 hours. The cultures were diluted 1:1000 into fresh LB, and cells were plated on LB agar plates containing kanamycin and ampicillin with l-arabinose supplement. Colonies were selected for PCR validation. Plasmids were subsequently eliminated by culturing the bacteria on LB plates containing sucrose at 37°C. Knockout mutants were confirmed by colony PCR with optimized primers.

### Fluorescence recovery after photobleaching (FRAP)

FRAP experiments were performed using an Olympus FV3000 confocal microscope. Condensates were bleached with a 405-nm Ar-laser pulse at maximum intensity. Data were analyzed using CellSens imaging software. ROIs were defined in the photobleached region, a nonphotobleached cell, and the background. The mean intensity of each was extracted with the correction of photobleach and background, and the fit FRAP curves were generated.

### In vitro protein expression and purification

Fusion proteins were expressed in *E. coli* BL21 and purified using a Ni-NTA column. Protein expression was induced overnight at 16°C with 0.5 mM IPTG. Cells were collected by centrifugation and resuspended in lysis buffer (20 mM tris-HCl, pH 7.4, 500 mM NaCl, and 10% glycerol). The suspension was sonicated for 30 min (2-s on and 4-s off, SCIENTZ) and centrifuged at 13,000*g* for 30 min at 4°C. The supernatant was incubated with Ni-NTA agarose for 20 min, washed, and eluted with 20 mM tris-HCl, pH 7.4, 500 mM NaCl, and 500 mM imidazole. Eluted proteins were stored in 20 mM tris-HCl, pH 7.4, 500 mM NaCl, and 1 mM dithiothreitol on ice or at −80°C.

### In vitro phase-separation assay

Purified DeaD protein was assembled by diluting the protein from a high-salt-storage buffer to varying salt concentrations (100 to 500 mM NaCl) and protein concentrations in 384-well plates (PhenoPlate). The DeaD-ECFP protein and SerRS^WT/T^-mCherry were gently mixed. After a 5-min incubation, images were captured with an Olympus FV3000 confocal microscope equipped with a ×40 objective.

To quantify the partition coefficient of SerRS^WT/T^ proteins by DeaD condensates, ROIs of equivalent size were analyzed in the Fiji implementation of ImageJ to calculate the SerRS signal intensity inside and outside DeaD condensates. The partition coefficient was defined as intracondensate fluorescence intensity divided by the fluorescence intensity of the extracondensate solution.

### tRNA charging assay

tRNA aminoacylation levels were analyzed using a periodate oxidation–based charging assay, as previously described (*89*). This method selectively oxidizes the 3′ ribose of uncharged tRNAs while leaving aminoacylated tRNAs protected by their covalently attached amino acids, enabling quantitative analysis through adaptor ligation and reverse transcription quantitative PCR.

Briefly, cells were harvested before (∆OD_600_ < 0.1) and after (∆OD_600_ ≥ 0.1) serine deprivation, chilled on ice, washed with cold PBS, and lysed in cold TRIzol (Invitrogen, 15596026). RNA was extracted by chloroform phase separation, followed by ethanol precipitation in the presence of GlycoBlue Coprecipitant (Invitrogen, AM9515). The RNA pellet was resuspended in 0.3 M sodium acetate buffer (pH 4.5) containing 10 mM EDTA, reprecipitated, and lastly dissolved in 10 mM sodium acetate buffer with 1 mM EDTA. For oxidation treatment, 2 μg of total RNA was incubated with 10 mM sodium periodate (“oxidized”) or 10 mM sodium chloride (“nonoxidized”) for 20 min at room temperature in the dark. Reactions were quenched with glucose, supplemented with yeast tRNA^Phe^ (Sigma-Aldrich, R4018) as the spike-in control, and precipitated with ethanol. RNA was then resuspended in 50 mM tris buffer (pH 9.0), incubated at 37°C for 50 min to deacylate any residual aminoacyl-tRNA, quenched with acetate buffer, and reprecipitated. Last, samples were resuspended in ribonuclease-free water and subjected to ligation to a 5′-adenylated DNA adaptor using truncated T4 RNA ligase 2 (NEB, M0373) at room temperature for 3 hours. Reverse transcription was carried out using SuperScript IV (Thermo Fisher Scientific, 18090050) and a primer complementary to the adaptor sequence (RT primer). cDNA was analyzed by quantitative PCR with isoacceptor-specific primers, where the forward primer annealed to the 5′ end of the tRNA and the reverse primer spanned the tRNA-adaptor junction. See table S3 for primer pairs used. Ct (cycle threshold) values for yeast tRNA^Phe^ were used for normalization. The aminoacylated (charged) tRNA fraction was calculated as the relative difference in ∆Ct (change in Ct) between oxidized (representing charged) and nonoxidized (total) samples.

### Aminoacylation activity assay

The in vivo aminoacylation activity assay of SerRS was determined using a modified method described by Cestari and Stuart (*90*). Reactions were performed in 50 μl of aminoacylation buffer containing 30 mM Hepes (pH 7.5), 140 mM NaCl, 30 mM KCl, and 40 mM MgCl_2_. Each reaction mixture included 1 mM dithiothreitol, 20 μM ATP, inorganic pyrophosphatase (2 U/ml), SerRS (40 μg/ml), l-serine, and tRNA^Ser^-CGA. Reactions were incubated at 37°C for 30 min and quenched by adding 10 mM EDTA on ice. Subsequently, 100 μl of malachite green solution was added to detect the release of inorganic phosphate, and absorbance was measured at 620 nm.

### Statistical analysis and data visualization

Sample sizes are selected as widely used in the field. Biological and technical replicates were performed as described in Materials and Methods for each experiment and conform to standards in the field. Two-tailed unpaired *t* tests (with Welch’s correction, where applicable), assuming a parametric distribution, were performed to assess differences across at least three replicates. *P* < 0.05 was considered significant. Statistical parameters including the definitions, exact values of *n*, and *P* values can be found in the figure legends and Materials and Methods. Data analysis was conducted using GraphPad Prism 10.2.0 software, and final figures were assembled with Adobe Illustrator.
