# Supplementary material for: DEAD-box ATPase–marked condensates coordinate compartmentalized translation and antibiotic persistence
Source: Sci Adv. 2026 Jan 2;12(1):eady1930. doi: 10.1126/sciadv.ady1930 (PMC12758526; doi:10.1126/sciadv.ady1930)
Supplement: Supplementary file 1 — Figs. S1 to S11 Tables S1 to S3 Legends for movies S1 and S2 [file sciadv.ady1930_sm.pdf]

Science Advances
AAAS

# Supplementary Materials for

## DEAD-box ATPase–marked condensates coordinate compartmentalized translation and antibiotic persistence

Ziyin Zhang *et al.*

Corresponding author: Jia-feng Liu, jfliu@mail.tsinghua.edu.cn

*Sci. Adv.* **12**, eady1930 (2026)
DOI: 10.1126/sciadv.ady1930

**The PDF file includes:**

Figs. S1 to S11
Tables S1 to S3
Legends for movies S1 and S2

**Other Supplementary Material for this manuscript includes the following:**

Movies S1 and S2

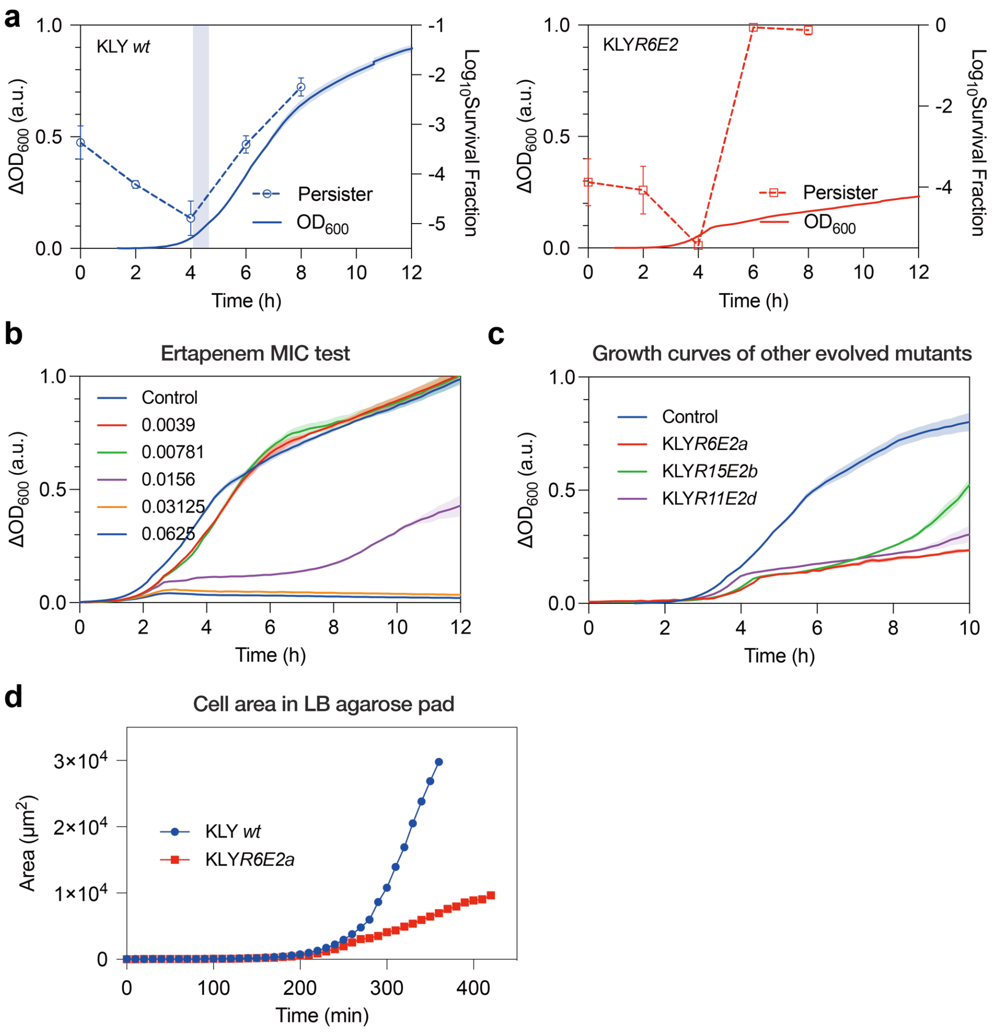


**Fig. S1 | Antibiotic persistence mediated by growth arrest**

(a) Growth curves and persister fractions for the ancestor (left) and *SerS*$^T$ tolerant (right) strains. Cultures were sampled at an interval of 2 hours to conduct the antibiotic killing assay. Shaded area, phase of periodical antibiotic exposure (*E. coli* KLY cultures) to evolve tolerant mutants. (b) MIC of ertapenem determined via microdilution. MIC values for *E coli KLY* ranged from 0.0156 to 0.03125 μg/mL. (c) Growth curves of the evolved isolates and the ancestral strains in LB medium. (d) Growth area measured over incubation time on LB agar pads, observed under a microscope, corresponding to Fig. 1f.

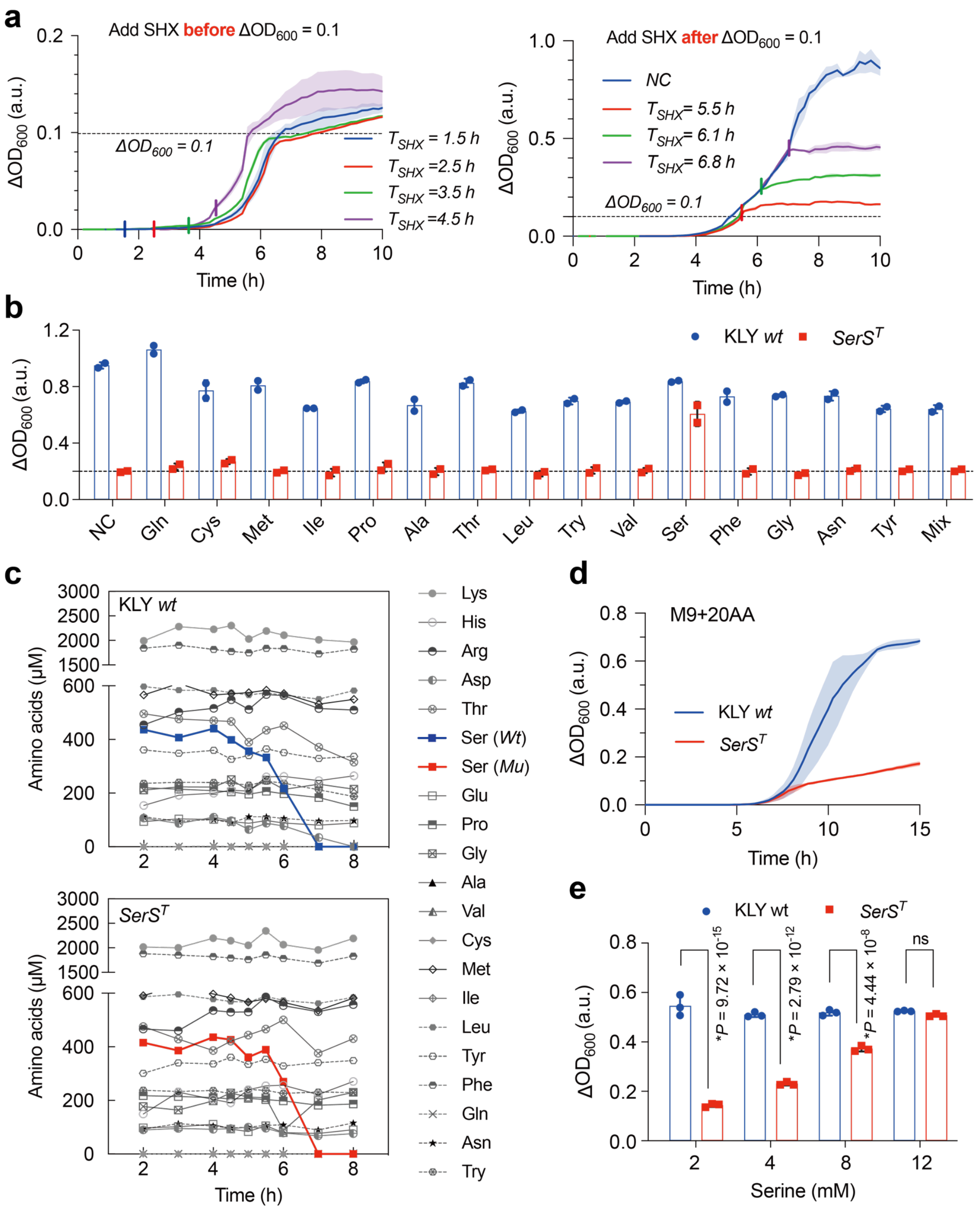


**Fig. S2 | Serine exhaustion triggered the growth-arrest mediated multidrug persistence**

(a) $OD_{600}$ measurements of KLY *wt* cultures exposed to SHX during growth in LB medium. Left panel, SHX (1 mM) was added at T=1.5, 2.5, 3.5, or 4.5 h, prior to serine deprivation ($\Delta OD_{600} < 0.1$). Right panel, SHX was added after serine depletion ($\Delta OD_{600} \geq 0.1$), as indicated by the colored solid lines. (b) Effect of various amino acid (AA) supplements on the growth defect of the mutant strain in LB medium. Each amino acid was added at a final concentration of 2 mM and cell densities were recorded after overnight incubation (~16 h). (c) Profiles of extracellular amino acid consumption by the ancestral and *SerS*$^T$ strains in LB medium. Data are derived from two biological replicates. (d) Growth defect of the *SerS*$^T$ strains in M9+20AA medium are comparable to that observed in LB medium. M9+20AA, a modified M9 medium augmented with all 20 amino acids, and each amino acid was added at 2 mM. (e) Growth of the ancestral and *SerS*$^T$ strains in M9+20AA medium, with serine selectively increased to 2, 4, 8, 12 mM. $OD_{600}$ measurements were recorded at stationary phase over 12 hours incubation.

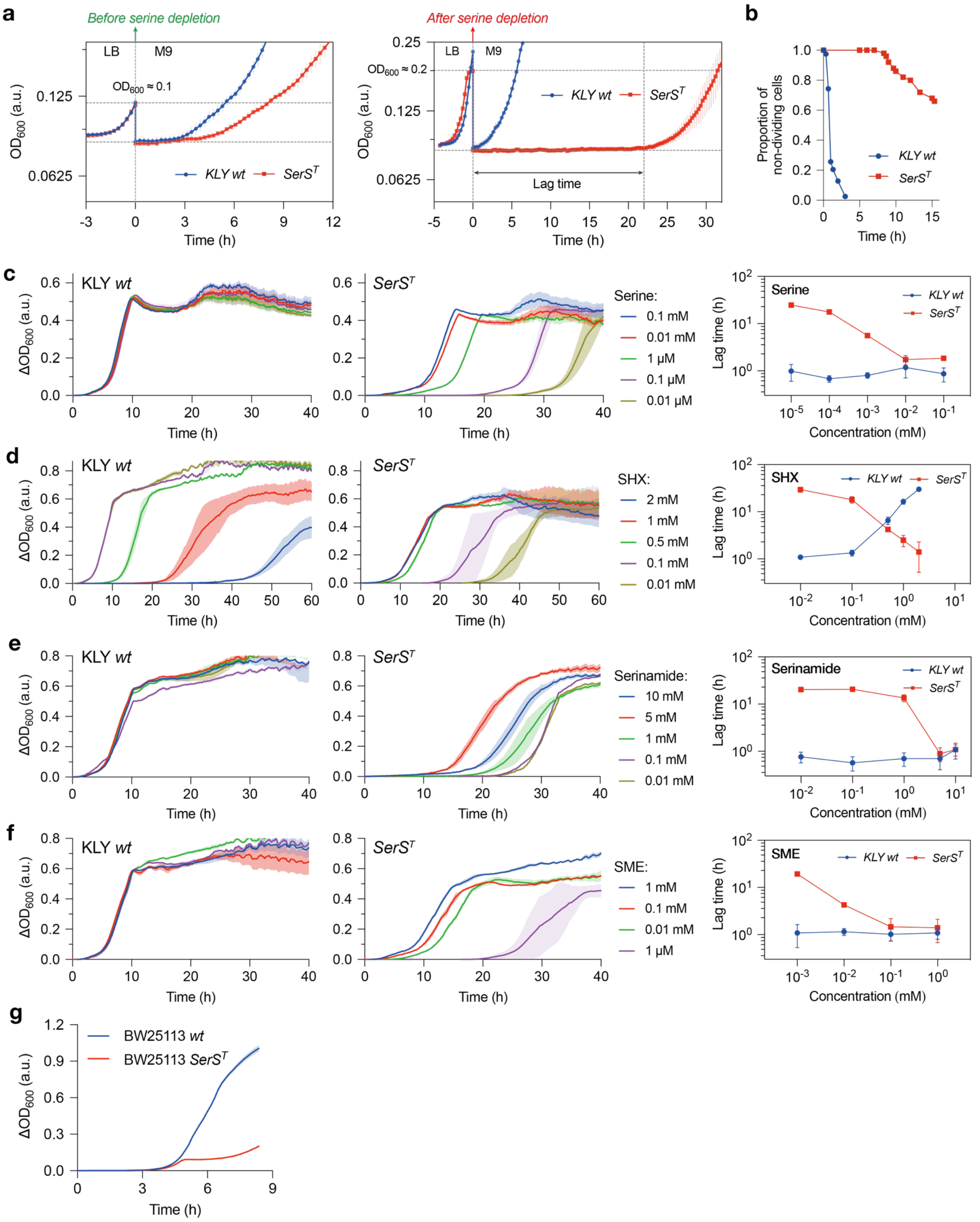


**Fig. S3 | Regulatory roles of serine and its analogues in bacterial dormancy**

(a) Lag times of wild-type KLY versus *SerS*$^T$ mutant in M9 medium, compared under conditions with (right) or without (left) serine deprivation stress. (b) Proportion of non-dividing cells over incubation time in M9 medium, related to Fig. 2d. (c-f) Growth curves of the ancestral and *SerS*$^T$ strains upon transfer to M9 medium, with varying concentrations of serine and its analogues. Lag time was determined as the time point at which $\Delta OD_{600}$ was less than 0.001, indicating minimal or no growth. (g) *SerS*$^T$ mutation recapitulates growth arrest in stringent response-functional *E. coli* BW25113 strain. Data are presented as the mean ± s.d. from three independent experiments.

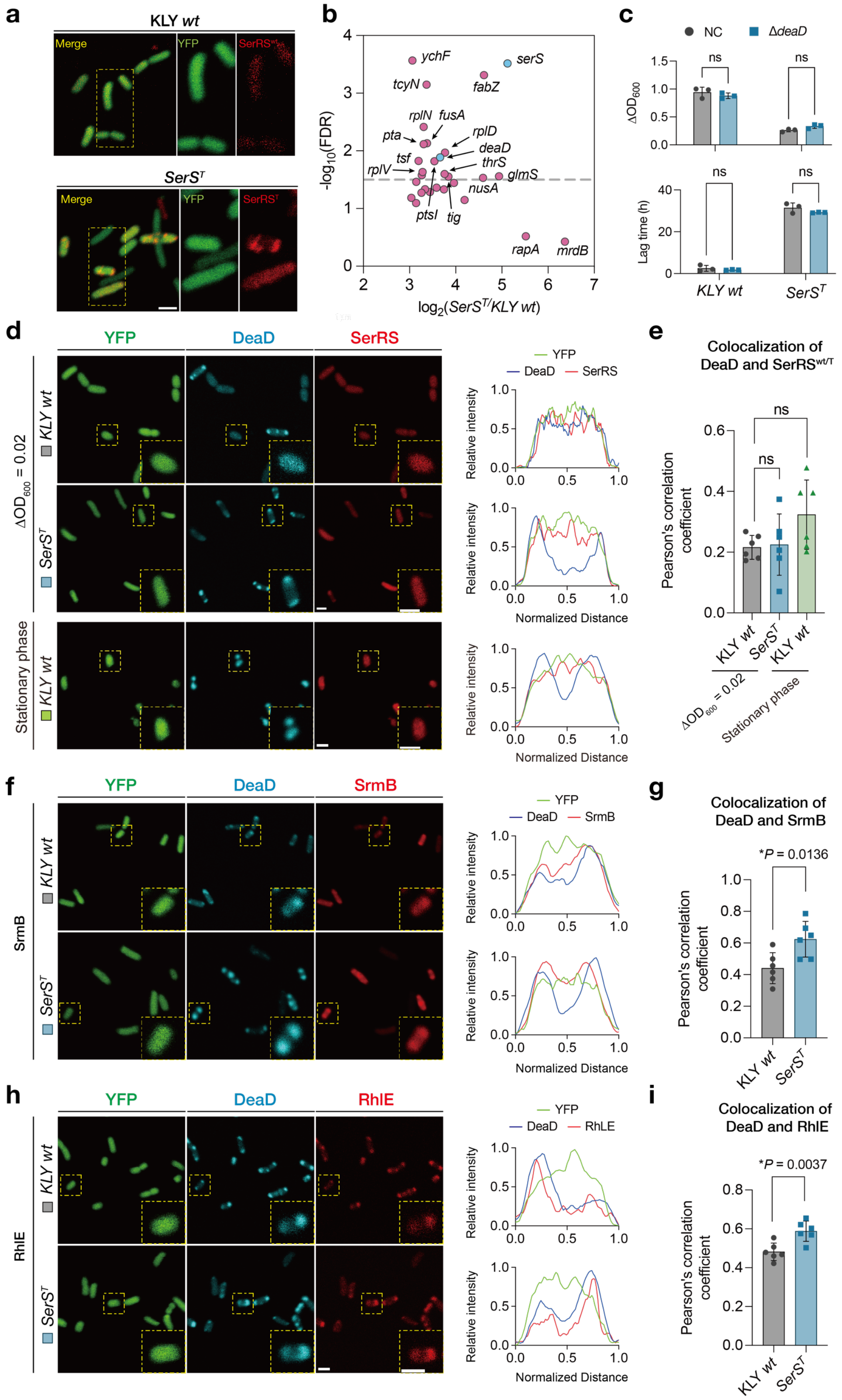


**Fig. S4 | DeaD-marked condensates sequestered multiple proteins potentially involved in the regulation of persistence**

(a) Fluorescence images of mCherry-tagged SerRS protein from *KLY wt* and the *SerS$^T$* cells, with an enlargement of yellow framed region on the right. Scale bar, 2 μm. (b) Candidates highly enriched in the soluble pellet fractions of the *SerS$^T$* strain relative to the ancestral KLY strain. Selection criteria: $\log_{10}$ *SerS$^T$* (protein abundance of insoluble condensates in the mutant)>8, $\log_2$ (*SerS$^T$*/KLY *wt*) (fold changes of the *SerS$^T$* strain over KLY strain) > 3., as indicated in the upper-right corner of Fig. 4b. (c) Cell density

in LB medium and lag time in M9 medium were measured in the ancestral and *SerS*$^{T}$ tolerant strains, both carrying a full-length knockout of the *deaD* gene. (d) Representative images of mCherry-tagged SerRS and ECFP-tagged DeaD in KLY wt and *SerS*$^{T}$ cells before serine deprivation ($\Delta OD_{600}$ < 0.1) and in the KLY *wt* cells during the stationary phase ($\Delta OD_{600} \approx 1.0$). Inset: higher magnification of the yellow boxed area. Scale bar, 2 μm. Line scans show the related intensity profiles of SerRS with DeaD signals, with Pearson's correlation coefficient indicated (e). (f, h) The colocalization of other DEAD-box ATPases, SrmB and RhlE, with DeaD-marked condensates in the ancestor and *SerS*$^{T}$ mutant strains. (g, i) Pearson's correlation coefficient showing the colocalization of DeaD-SrmB (g) and DeaD-RhlE (i). Data are presented as the mean ± s.d. with a two-tailed t-test.

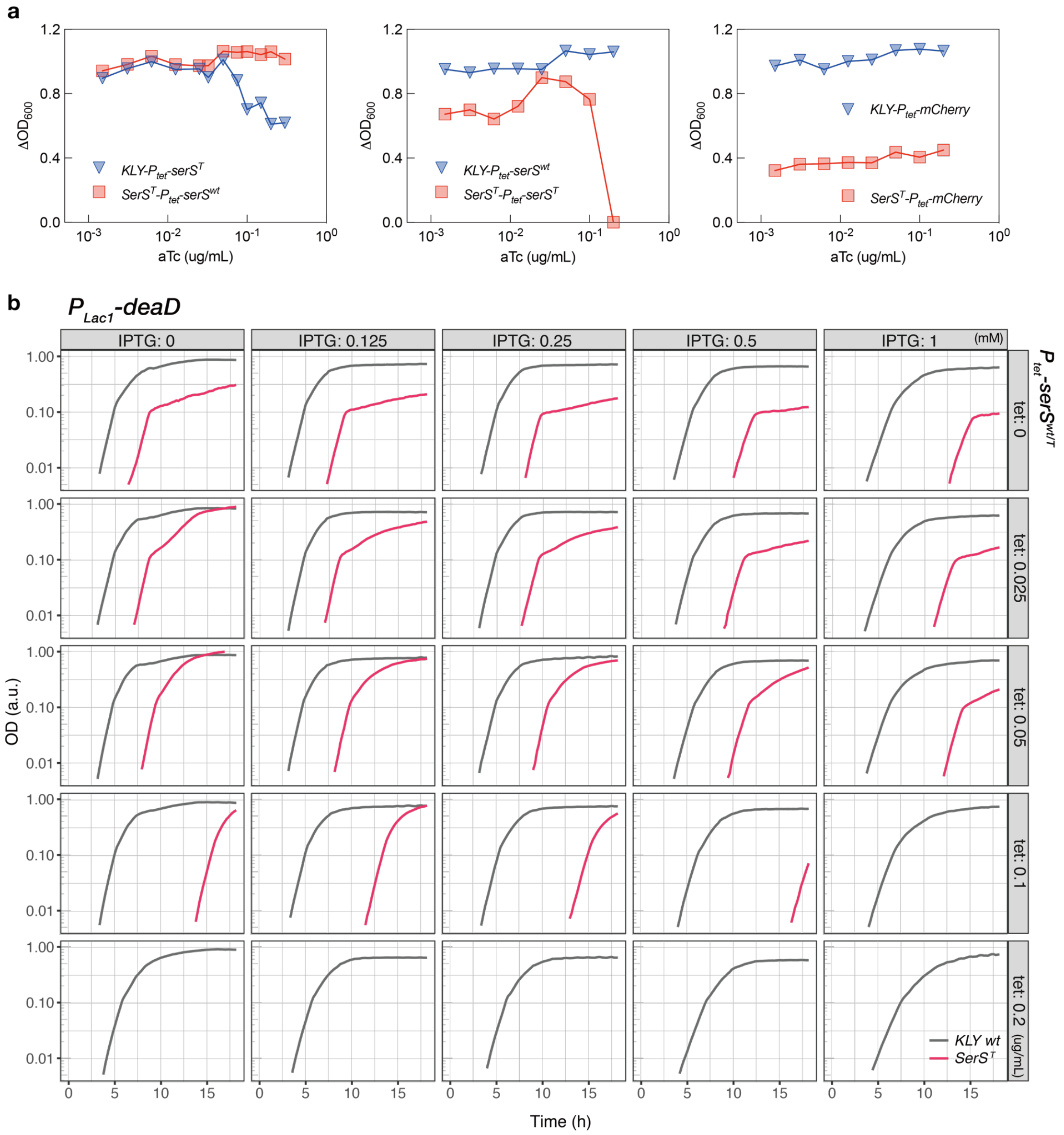


**Fig. S5 | DeaD and SerRS$^T$ function as regulators of growth dynamics with opposing effects**
(a) Cell growth recovery in the *SerS$^T$* tolerant strain by different levels of tetracycline. (b) OD measurement over incubation time with the different expression levels of DeaD and SerRS in the ancestral KLY and *SerS$^T$* strains in LB medium. The KLY *wt* and *SerS$^T$* strain co-expressed the DeaD and the respective *serS$^{wt}$* or *serS$^T$* gene, induced by varying levels of IPTG and tetracycline.

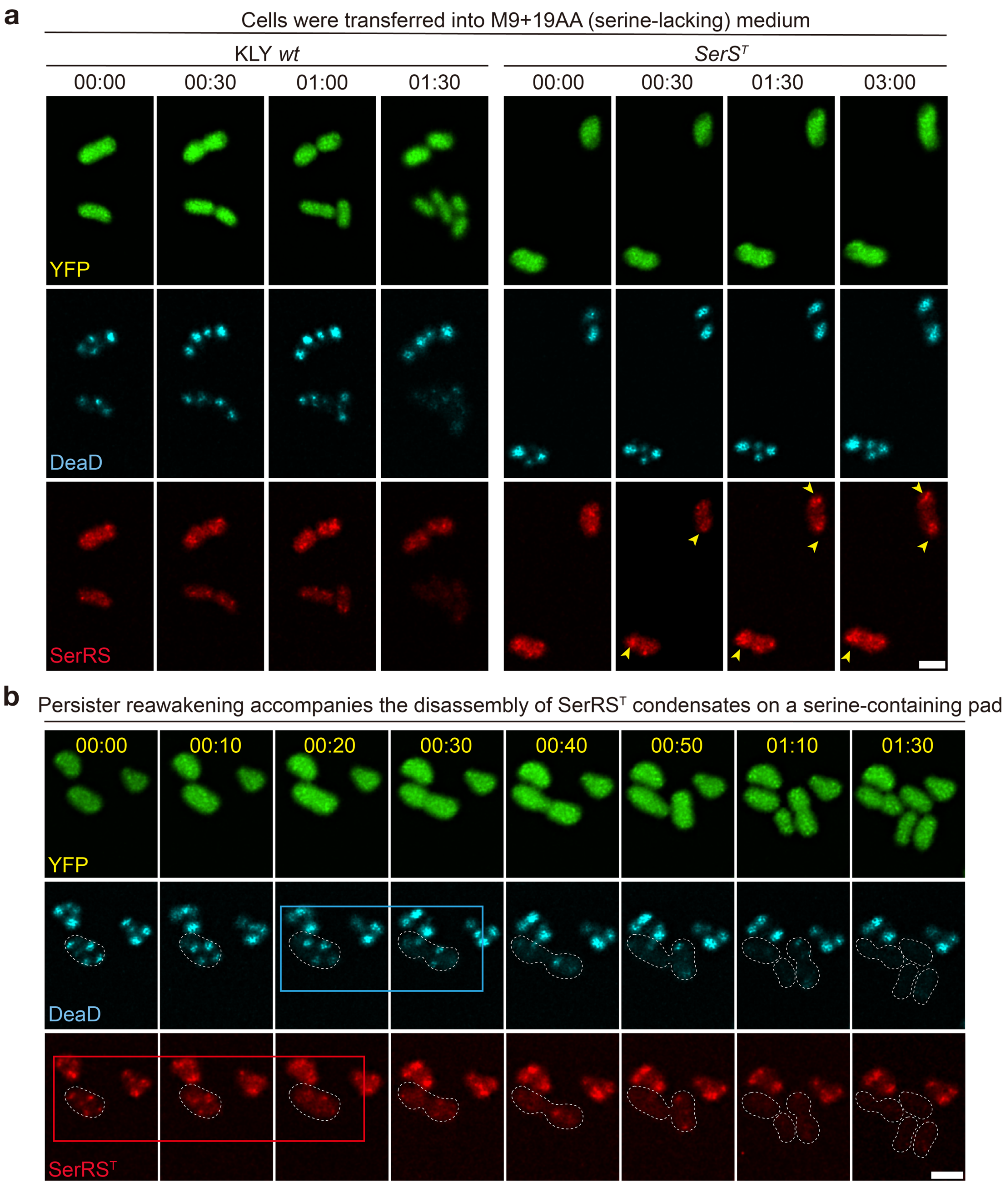


**Fig. S6 | SerRS$^T$-DeaD condensates mediate reversible phenotypic switches in response to serine availability**

(a) Assembly of SerRS$^T$-DeaD condensates upon serine deprivation. Time-lapse microscopy of KLY *wt* and *SerS$^T$* mutant cells following transfer from serine-replete LB medium to the serine-depleted M9+19AA medium (M9+19 amino acids except serine, each at 2 mM). Time points indicate hours:minutes post-transfer. Yellow arrows highlight SerRS$^T$-DeaD condensate formation. Scale bar, 2 μm. (b) Persister resuscitation coincides with condensate disassembly. Time-lapse microscopy of *SerS$^T$* persisters, isolated from ertapenem treatment, recovering on M9 agarose pad supplemented with 0.1 mM serine. Red box, release of SerRS$^T$ protein from the DeaD-marked condensates. Blue box, initiation of DeaD condensates structural disassembly. Time points indicate hours:minutes post-seeding. Scale bar, 2 μm. Note: In both panels, fluorescence intensity decreases in actively dividing cells due to dilution of pre-induced SerRS-mCherry and DeaD-ECFP, while stable fluorescence in growth-arrested cells reflects reduced proliferation, enabling fluorescence dilution to serve as an indicator of cell division status.

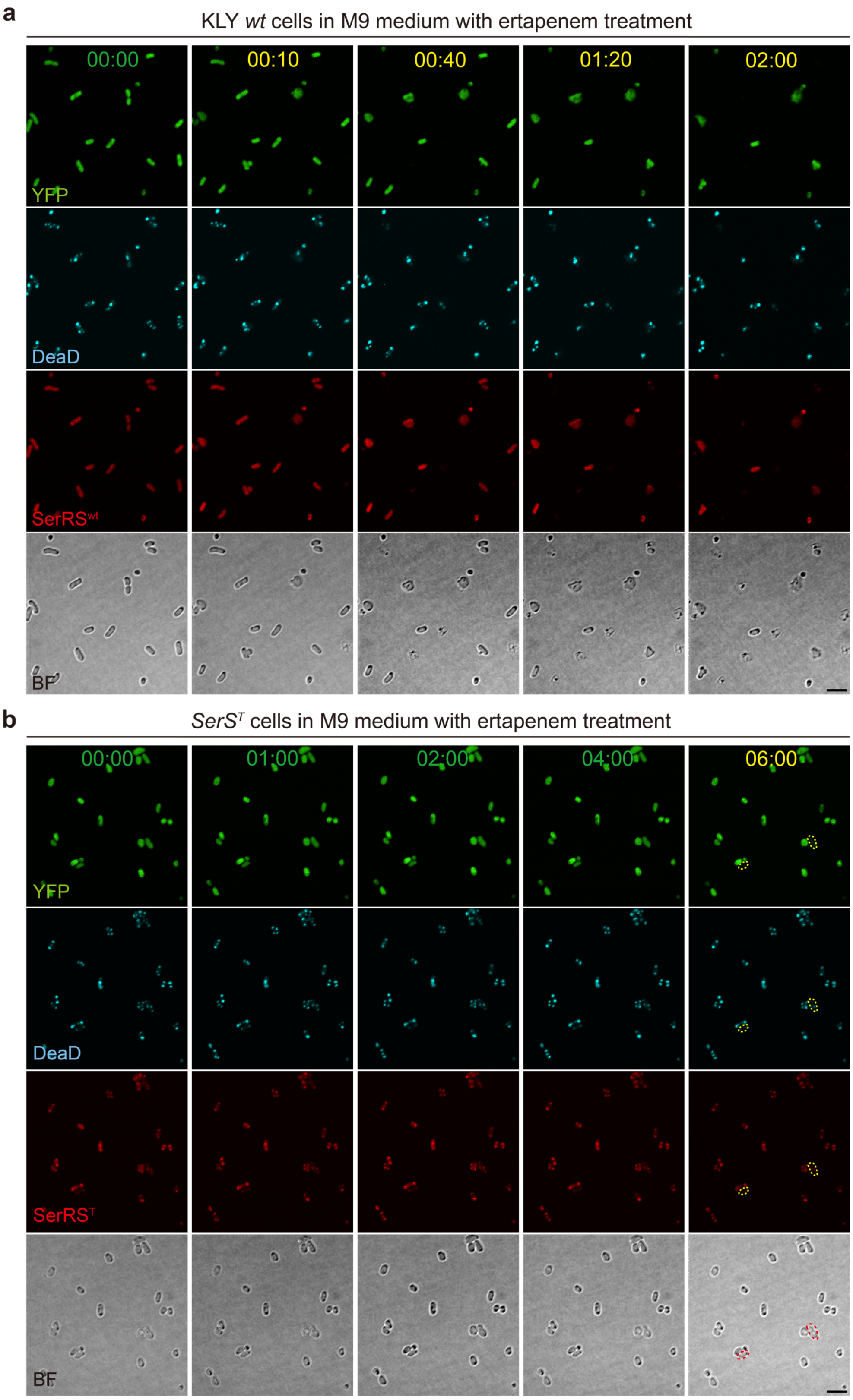


**Fig.S7 | Time-resolved cell death dynamics under antibiotic exposure**

KLY *wt* (a) and *SerS$^T$* (b) cells were precultured in LB medium under conditions that induced expression of SerRS-mCherry and DeaD-ECFP, and grown until serine was depleted. The cultures were transferred to M9 medium containing lethal-dose ertapenem (5 μg/mL, >100 × MIC). Images were taken at indicated time points post-antibiotic exposure. Dashed outlines highlight lysed cells in the *SerS$^T$* strain, while the majority of KLY *wt* cells were killed with 2 hours of treatment. Scale bar, 5 μm.

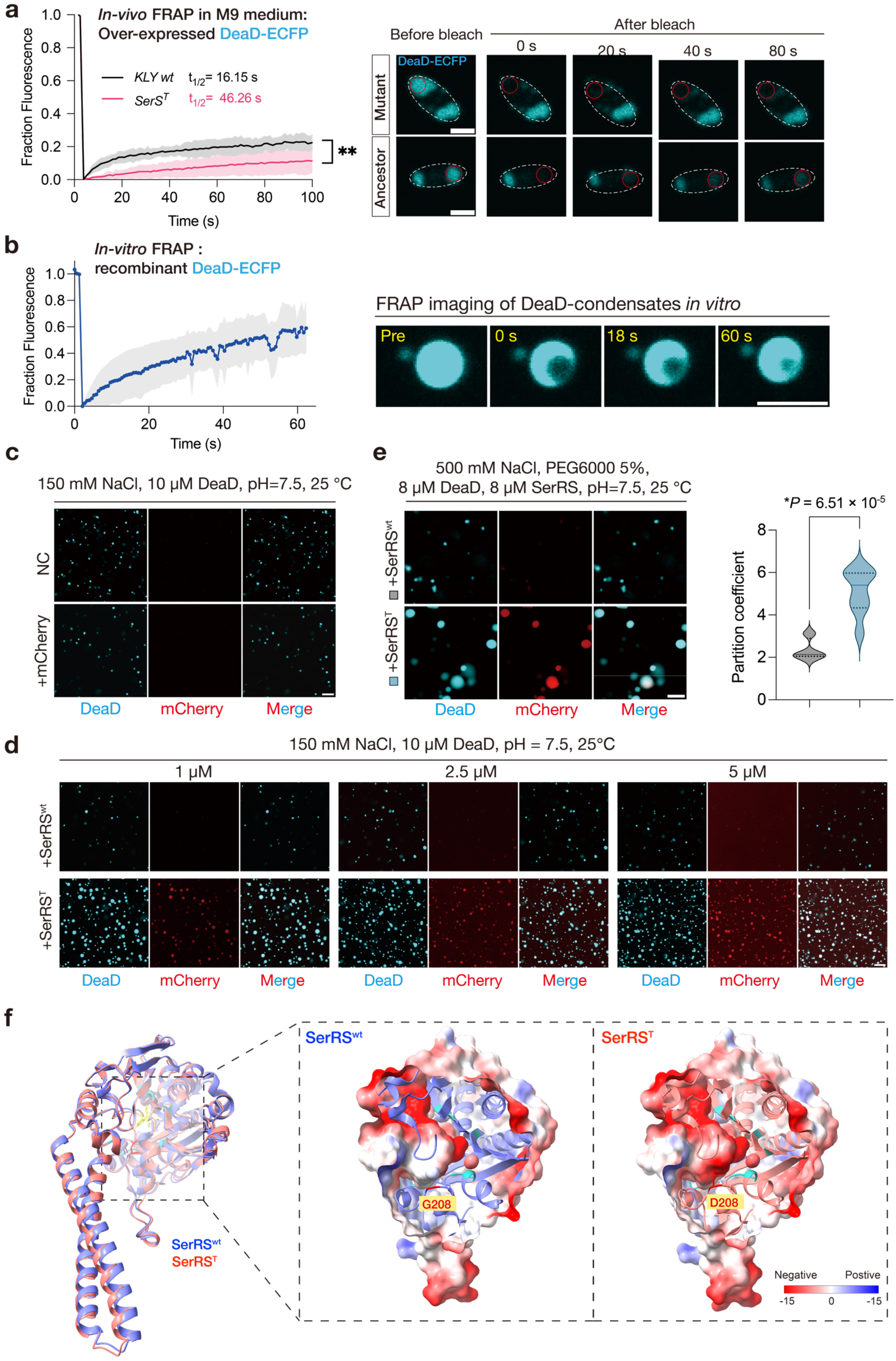


**Fig. S8 | DeaD undergoes phase separation to sequester SerRS$^T$**

(a) FRAP analysis of DeaD-granules in the ancestral KLY and *SerS$^T$* mutant cells cultured in M9 medium. Data are presented as mean ± s.d.; **p < 0.01 by two-way ANOVA with Sidak's multiple comparison test. Mobile fractions were quantified at 25% (KLY *wt*), 18% (*SerS$^T$*). Scale bar, 1 μm. (b) FRAP analysis of DeaD droplets *in vitro*. Error bars indicate s.d.; n = 6 condensates. Scale bar, 5 μm. (c) LLPS of purified recombinant DeaD in the presence of 1 μM mCherry in buffer containing 150 mM NaCl without PEG.

Scale bar, 10 μm. (d) LLPS of purified recombinant DeaD with increasing concentrations of $SerRS^{wt}$ or $SerRS^{T}$ proteins in buffer containing 150 mM NaCl without PEG. Scale bar, 10 μm. (e) $SerRS^{T}$ exhibited a comparable translocation pattern in DeaD-marked condensates formed with PEG. Data are mean ± s.d.; *P* values were calculated using two-tailed t-tests with Welch's correction; n = 8 condensates, respectively. Scale bar, 10 μm. (e) $SerRS^{T}$ exhibited a comparable translocation pattern in DeaD condensates formed with PEG. Data are mean ± s.d.; *P* values were calculated using two-tailed t-tests with Welch's correction; n = 8 condensates. Scale bar, 10 μm. (f) Cut-open views showing the electrostatic surface distribution of $SerRS^{wt}$ and $SerRS^{T}$. Color scale ranges from negative (red) through neutral (white) to positive (blue). The $SerRS^{wt}$ structure complexed with seryl sulfamoyl adenylate (Ser-SA, yellow) was obtained from Protein Data Bank (PDB ID: 6R1M). The $SerRS^{T}$ structure (G208D) was predicted by AlphaFold 3. Visualization was performed with ChimeraX-1.10.1.

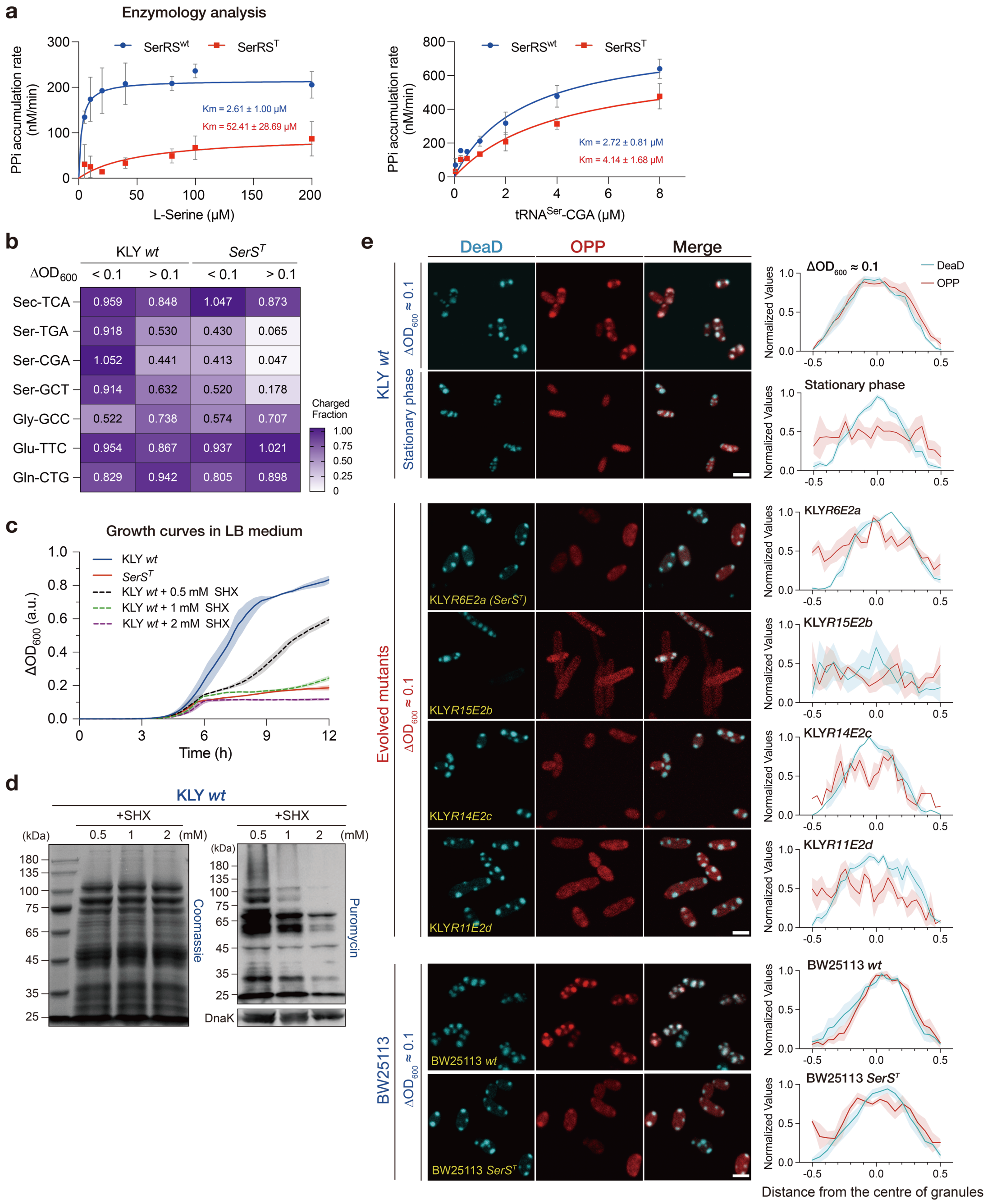


**Fig. S9 | Condensate-localized translational silencing unifies diverse persistence states**

(a) Michaelis–Menten kinetics of $SerRS^{wt}$ and $SerRS^{T}$ variant. Reactions were performed with varying concentrations of the cognate substrate L-serine (5–200 μM) in the presence of 1 μM $tRNA^{Ser}$-CGA and 40 μg/mL purified SerRS (left), or with a fixed concentration of L-serine (200 μM) and varying levels of $tRNA^{Ser}$-CGA (0.05–8 μM) (right). All assays were carried out at 37°C for 30 min. (b) KLY *wt* and $SerS^{T}$ cells were sampled before ($\Delta OD_{600} < 0.1$) and after ($\Delta OD_{600} \geq 0.1$) serine depletion, followed by in vivo tRNA charging analysis. (c) Growth curves of KLY *wt* in LB medium when exposed to different concentrations of SHX in LB medium. (d) Western blot of puromycin-labeled nascent polypeptides in SHX-treated KLY *wt* cells upon growth arrest (labeling time: 30 min). These SHX-treated translation patterns phenocopied the $SerS^{T}$ mutant (relative to Fig. 5a). (e) Co-localization of nascent peptides and DeaD-marked condensates across physiological states (stationary phase), evolved mutants (KLY*R15E2b*, KLY*R14E2c*, KLY*R11E2d*) and the BW25113$SerS^{T}$ strain. Signal intensity profiles of OPP-labelled nascent polypeptides (red), centered at DeaD-marked condensates (cyan). Shaded areas around lines indicate SEMs (n=6). Scale bar, 2 μm.

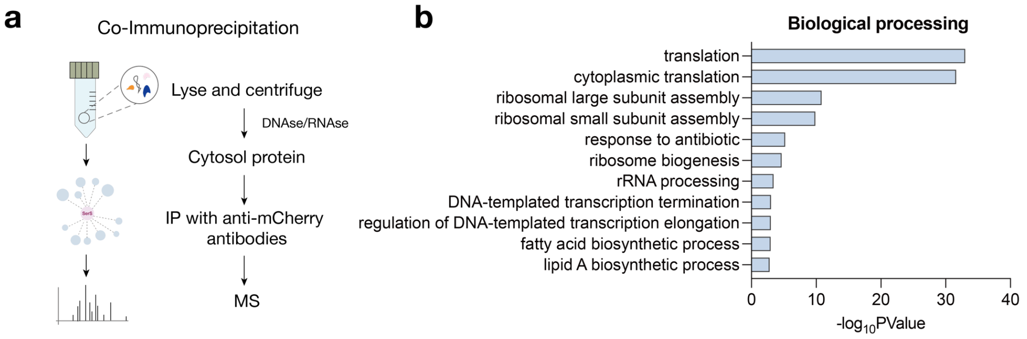


c

| | | | | | | | | | | |
|---|---|---|---|---|---|---|---|---|---|---|
| Proteins Putatively Co-precipitating with Cytosolic SerRS$^T$ | Ribosome subunits (27) | rplW | rpsG | rpsS | rplQ | rpsD | rpsA | rpsH | deaD | rpsJ |
| | Translation related factors (7) | efp | ettA | fusA | infB | infC | prfB | prfC | | |
| | AARS (6) | glyQ | glyS | hisS | lysS | pheT | proS | | | |
| | rRNA processing (5) | hrpA | rne | rluC | rluB | rlmN | | | | |
| | Ribosomal assembling factors (9) | der | era | obgE | rimM | rlmE | bipA | uup | yhbY | yheS |

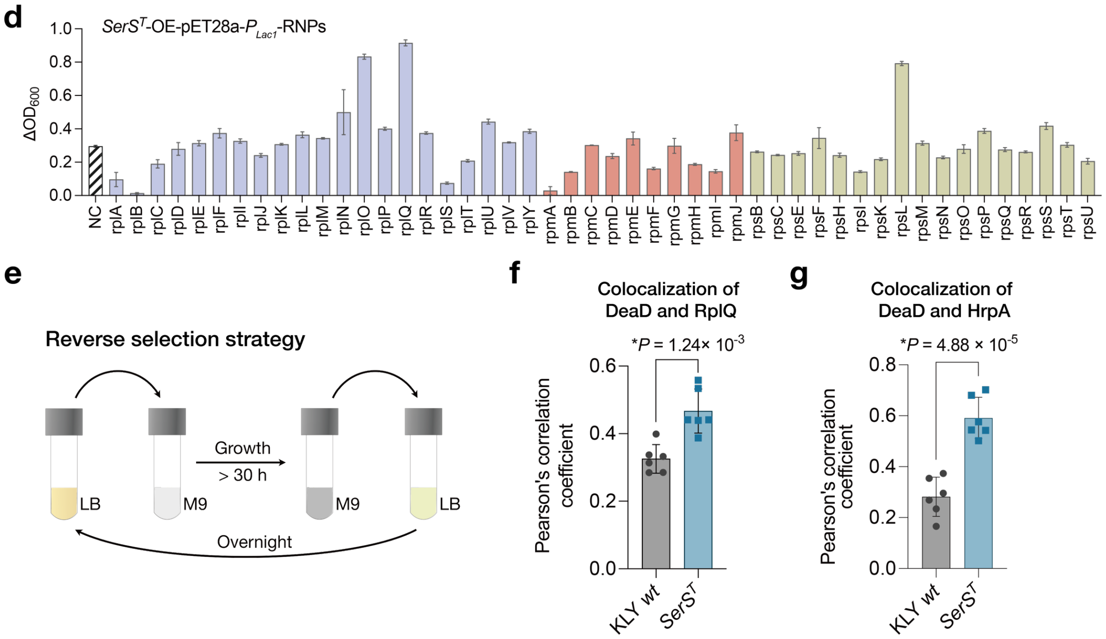


**Fig. S10 | Altered composition of condensates in the *SerS$^T$* mutant compared to the KLY strain**

(a) Schematic of the experimental workflow, including cellular fractionation, immunoprecipitation, and LC-MS/MS analysis to identify SerRS-interacted proteins in the cytosol. (b) GO analysis of proteins immunoprecipitated from the SerRS$^T$ versus SerRS$^{wt}$ interactomes. The top 10 GO biological process annotations were shown as a bar chart, ranked by frequency (top to bottom), with the horizontal axis representing the -log10 *P* values of each term. (c) List of proteins identified by LC-MS/MS. (d) Effect of ribonucleoprotein (RNP) overexpression on the recovery of growth defects in the *SerS$^T$* tolerant strain grown in LB medium. Purple and orange bars denote proteins of the 50S ribosomal subunit, while light green bars correspond to the 30S ribosomal subunit. (e) Schematic of the reverse selection experimental design. (f, g) Pearson's correlation coefficients for colocalization of DeaD with RplQ (f), and DeaD with HrpA (g). Data are presented as the mean ± s.d.; *P* values were calculated using two-tailed t-tests.

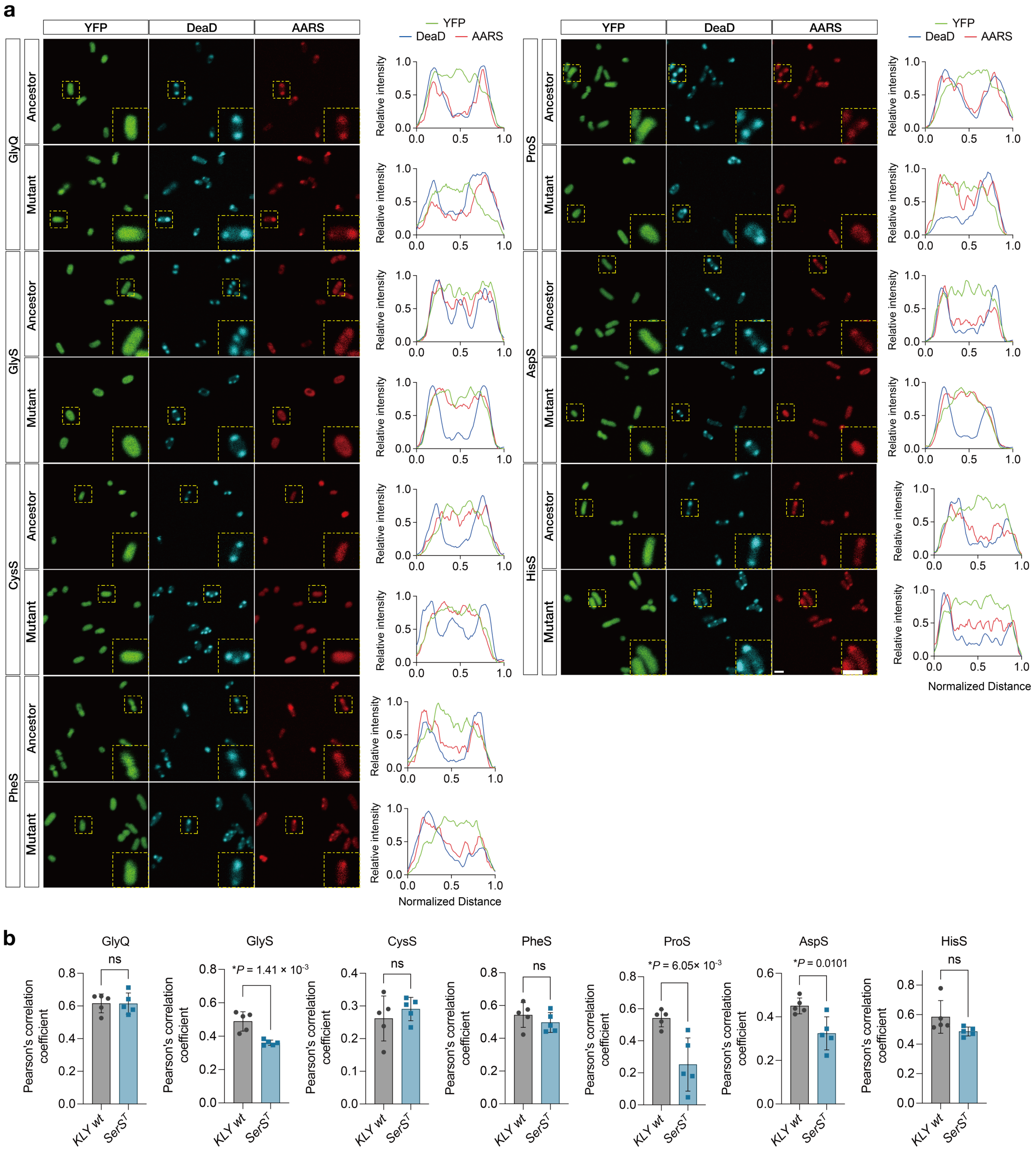


**Fig. S11 | Altered colocalization of aaRSs and DeaD-marked condensates**

(a) Representative fluorescence images of mCherry-tagged aaRSs in cells expressing ECFP-tagged DeaD in the KLY *wt* and $SerS^T$ mutant cells. Inset: higher magnification of the yellow boxed area. Line scans show the related intensity profiles of aaRSs with DeaD. Scale bar, 2 μm. (b) Pearson's correlation coefficients for colocalization analysis. Data are represented as mean ± s.d.; *P* values were calculated using two-tailed t-test.

**Table S1. Main mutations detected in whole-genome sequencing data and verified by Sanger sequencing**

| Strain | Locus | Amino acid substitution | Gene | Annotation |
|---|---|---|---|---|
| **KLY R6E2a (*SerS$^T$*)** | 939,081 | G208D (GGT -> GAT) | *serS* | Seryl-tRNA synthetase |
| **KLY R15E2b** | 939,066 | T203R (ACG -> AGG) | *serS* | Seryl-tRNA synthetase |
| **KLY R14E2c** | 553,976 | P112L (CCG -> CTG) | *cysS* | cysteinyl-tRNA synthetase |
| | 3,858,644 | M256I (ATG -> ATA) | *cysE* | Serine acetyltransferase |
| | 3,563,333 | N66K (AAT -> AAG) | *crp* | cAMP-activated transcriptional regulator |
| **KLY R11E2d** | 939,303 | I282S (ATC -> AGC) | *serS* | Seryl-tRNA synthetase |

**Table S2. The plasmids used in this study**

| Name | Source |
|---|---|
| p15A-$P_{tet}$-DeaD ($Kan^R$) | This paper |
| p15A-$P_{tet}$-DeaD-His ($Kan^R$) | This paper |
| p15A-$P_{tet}$-$SerRS^{wt}$ ($Kan^R$) | This paper |
| p15A-$P_{tet}$-$SerRS^{wt}$-mCherry ($Kan^R$) | This paper |
| p15A-$P_{tet}$-$SerRS^{T}$ ($Kan^R$) | This paper |
| p15A-$P_{tet}$-$SerRS^{T}$-mCherry ($Kan^R$) | This paper |
| p15A-$P_{tet}$-HisS-mCherry ($Kan^R$) | This paper |
| p15A-$P_{tet}$-GlyS-mCherry ($Kan^R$) | This paper |
| p15A-$P_{tet}$-GlyQ-mCherry ($Kan^R$) | This paper |
| p15A-$P_{tet}$-AspS-mCherry ($Kan^R$) | This paper |
| p15A-$P_{tet}$-ProS-mCherry ($Kan^R$) | This paper |
| p15A-$P_{tet}$-CysS-mCherry ($Kan^R$) | This paper |
| p15A-$P_{tet}$-PheS-mCherry ($Kan^R$) | This paper |
| p15A-$P_{tet}$-HrpA ($Kan^R$) | This paper |
| p15A-$P_{tet}$-HrpA-mCherry ($Kan^R$) | This paper |
| p15A-$P_{tet}$-SrmB-mCherry ($Kan^R$) | This paper |
| p15A-$P_{tet}$-RhlE-mCherry ($Kan^R$) | This paper |
| p15A-$P_{tet}$-ObgE-mCherry ($Kan^R$) | This paper |
| pET28a-$P_{lac1}$-DeaD ($Amp^R$) | This paper |
| pET28a-$P_{lac1}$-DeaD-ECFP ($Amp^R$) | This paper |
| pET28a-$P_{lac1}$-RBPs-mCherry ($Kan^R$) [a] | This paper |
| p15A-$P_{BAD}$-Cas9-$P_{T5}$-Red$\gamma\beta\alpha$ | Yi-xin Huo Provided (Beijing Institute of Technology) |
| $P_{BAD}$-sgRNA-Donor [b] | Yi-xin Huo Provided (Beijing Institute of Technology) |

Note:

a. all the ribosomal subunits were respectively overexpressed in this plasmid.
b. pBAD-sgRNA-Donor containing different editing fragments

**Table S3. Primers used in tRNA charging assay**

| Primers | Sequence |
|---|---|
| tRNA Adaptor | 5'-/5rApp/TGGAATTCTCGGGTGCCAAGG/3ddC/-3' |
| RT primer | GCTGCCTTGGCACCCGAGAATTCCA |
| Sec-TCA | F: GGAAGATCGTCGTCTCCGG<br>R: GAGAATTCCATGGCGGAAGATC |
| Ser-CGA | F: GGAGAGATGCCGGAGCG<br>R: GAGAATTCCATGGCGGAGAGAG |
| Ser-GCT | F: GGTGAGGTGKCCGAGWGGC<br>R: GAGAATTCCATGGCGGTGAGGS |
| Ser-TGA | F: GGAAGTGTGGCCGAGCGG<br>R: GAGAATTCCATGGCGGTGAGGS |
| Gly-GCC | F: GCGGGAATAGCTCAGTTGGTAGAGC<br>R: GAGAATTCCATGGAGCGGGAAACG |
| Glu-TTC | F: GTCCCCTTCGTCTAGAGGCCC<br>R: GAGAATTCCATGGCGTCCCCTAGG |
| Gln-CTG | F: TGGGGTATCGCCAAGCGG<br>R: GAGAATTCCATGGCTGGGGTACS |

## Other Supplementary Material for this manuscript includes the following:

Movie S1 Time-lapse microscopy of single bacteria plated on fresh LB medium
Movie S2 Time-lapse microscopy of single bacteria transferred from LB cultures to M9 medium.
